# Fatal Congenital Toxoplasmosis with Progressive Liver Failure and Genomic Characterization of a Novel Isolate from the United States

**DOI:** 10.3390/microorganisms13122865

**Published:** 2025-12-17

**Authors:** Katsuaki Kojima, Indu Varier, Rouba Sayegh, Masako Shimamura, Bimal P. Chaudhari, Anas Bernieh, Matthew J. Schulz, Peter White, James Fitch, Alexandra K. Medoro, Hernan A. Lorenzi, Rima McLeod

**Affiliations:** 1 Cincinnati Children’s Hospital Medical Center, University of Cincinnati College of Medicine, Cincinnati, OH 45229, USA; 2 Nationwide Children’s Hospital, The Ohio State University, Columbus, OH 43215, USA; rouba.sayegh@hsc.wvu.edu (R.S.);; 3 National Institute of Diabetes and Digestive and Kidney Diseases, National Institutes of Health, Phoenix, AZ 85016, USA; hernan.lorenzi@nih.gov; 4 Departments of Ophthalmology and Visual Sciences and Pediatrics (Infectious Diseases), The University of Chicago, Chicago, IL 60637, USA

**Keywords:** congenital toxoplasmosis, disseminated toxoplasmosis, liver failure, prenatal screening

## Abstract

Congenital toxoplasmosis presents with a wide clinical spectrum, ranging from asymptomatic infection to severe disease with multi-organ failure. We report a rare fatal case of disseminated congenital toxoplasmosis in a human neonate. The infant initially had thrombocytopenia and mild hepatitis, which rapidly progressed to fulminant liver failure. Despite initiation of standard therapy with pyrimethamine, sulfadiazine, and folinic acid on postnatal day 25, the infant died two days later. Autopsy revealed widespread involvement of the liver, spleen, brain, heart, lungs, urinary bladder, and skeletal muscle. To further characterize the infection, genomic sequencing of the isolate (TgHsUS2) was performed, which placed it within clade C (Haplogroup 9) and closely related to reference strains P89 and TgCatBr3. Variant analysis showed type III-like alleles in ROP18, ROP16, and GRA15. These alleles are known to modulate host immunity and may have influenced disease severity in this case. This report highlights the need for rapid recognition and targeted therapy as well as how strain genomics can inform disease mechanisms. Prevention through prenatal screening and maternal treatment during pregnancy may reduce infant mortality.

## 1. Introduction

Congenital toxoplasmosis, caused by the pervasive protozoal parasite *Toxoplasma gondii* (*T. gondii*), manifests with a range of symptoms in fetuses and newborns [1,2,3]. The infection occurs in the fetus typically when a pregnant woman contracts an acute primary infection or, less frequently, when she undergoes a reactivation of toxoplasmosis [4,5]. The seroprevalence of this infection varies widely, from a few percent to over 50% in some communities [6,7]. Many women of childbearing age are seronegative [2,8]. If they acquire a primary *T. gondii* infection during pregnancy, fetal transmission can occur [4,5,9]. Recognition and prevalence vary widely across countries [10] and within the United States (US) [11].

Among neonates diagnosed with congenital toxoplasmosis in the US from 1991 to 2005, 92% had chorioretinitis, 80% exhibited intracranial calcifications, and 68% had hydrocephalus [12]. In total, 62% presented with all three conditions, often described as the classic triad of congenital toxoplasmosis [2,12]. These data require caution. They come from a referral database that likely over-represents severe cases. In the National Collaborative Toxoplasmosis Study (NCCCTS), children with milder symptoms or none have been referred, especially infants treated in utero [13,14].

While the ocular and neurologic manifestations of congenital toxoplasmosis are well described, disseminated disease characterized by septic shock and multi-organ failure is much less frequently recognized [1]. Underreporting has several causes [3]. First, early signs (e.g., thrombocytopenia, hepatitis, respiratory distress) are nonspecific [1,9]. Second, critically ill neonates may not receive advanced imaging or specialized serology early. Third, routine maternal screening is not standard in the US [2]. As a result, severe disseminated presentations may be misattributed to bacterial sepsis or other causes of neonatal liver failure, leading to delayed or missed diagnosis [15]. Greater awareness of these atypical and severe presentations is needed to guide earlier consideration of *T. gondii* in the differential diagnosis of critically ill neonates with multi-organ involvement.

## 2. Case Presentation and Genomic Characterization

### 2.1. Case Presentation

#### 2.1.1. Maternal and Perinatal History

We describe a neonate with fatal disseminated congenital toxoplasmosis, rapidly progressive liver failure, and multi-organ involvement. A male infant was born at 38 weeks’ gestation to a 24-year-old mother who was gravida 3, para 2 (three pregnancies, two prior live births), with no known consanguinity with the father. Maternal prenatal testing showed blood type A+, negative antibody screening (indicating absence of red blood cell alloantibodies), hepatitis B surface antigen negativity, syphilis IgG nonreactivity twice, HIV IgG negativity twice, rubella immunity, and vaginal culture positive for *Streptococcus agalactiae*. Maternal prenatal *T. gondii* serologic testing was not performed because routine screening is not recommended in US obstetric practice [16].

The family had recently moved from Alabama to Ohio and lived in an apartment, owning a parakeet but no cats. Neighbors had outdoor cats, but the mother reported no contact with them and did not care for any cats during pregnancy. She denied febrile illnesses during pregnancy. She denied consuming wild game, oysters, and unpasteurized dairy but did occasionally eat rare steak. The family did not drink unpasteurized milk, and the mother had not received an organ transplant or blood transfusion. She denied travel to Australia, South America, or Africa. The only potential environmental exposure was occasional visits with her toddlers to a local playground sandbox. The delivery was carried out via an emergency cesarean section due to non-reassuring fetal heart tones and a history of prior cesarean sections.

#### 2.1.2. Neonatal Presentation

The newborn’s birth weight was 2680 g (7th percentile), and his head circumference measured 32.5 cm (7th percentile). His Apgar scores were 7 and 8 at 1 and 5 min after birth, respectively. Initial clinical examination did not reveal hepatosplenomegaly.

Key laboratory findings included transient hypoglycemia (<10 mg/dL), persistent thrombocytopenia (16,000/mm^3^), elevated direct bilirubin (1.9 mg/dL; reference range < 0.6 mg/dL), and elevated aspartate aminotransferase (AST) (125 U/L; reference range 20–60 U/L). Due to respiratory distress, the infant required non-invasive respiratory support.

Multiple platelet transfusions and intravenous immunoglobulin (IVIG) at 1 g/kg/dose for 3 doses were given for refractory thrombocytopenia to treat possible neonatal alloimmune thrombocytopenia (NAIT) per hematology consultation. NAIT was subsequently excluded based on negative maternal anti-platelet antibody testing.

#### 2.1.3. Diagnostic Workup

Given the thrombocytopenia and mildly abnormal liver enzymes, congenital and perinatal infections were included in the differential diagnosis. Testing for congenital Cytomegalovirus (CMV) infection by saliva and urine polymerase chain reaction (PCR) was negative. Blood PCR testing was negative for Herpes Simplex Virus (HSV), Epstein–Barr Virus (EBV), Enterovirus, Parechovirus, adenovirus, parvovirus B19, and hepatitis C. Hepatitis A and E serologies and the hepatitis B surface antigen were also negative. Both blood and urine cultures were negative for bacterial pathogens. An abdominal ultrasound revealed no hepatosplenomegaly, and a cephalic ultrasound showed no hydrocephalus or calcification.

*T. gondii* serologies were performed at ARUP Laboratories (Salt Lake City, UT, USA), a national reference laboratory that provides specialized diagnostic testing, including standardized enzyme immunoassays for *T. gondii*. These tests, drawn on postnatal day 6 after receiving IVIG, showed elevated IgG (37 IU/mL; reference range ≤ 7.1 IU/mL) and negative IgM (3.5 AU/mL; reference range ≤ 7.9 AU/mL).

On postnatal day 15, the infant developed fever, abdominal distension, and leukocytosis. Nafcillin and gentamicin were started for suspected late-onset sepsis. On day 16, an infectious disease consultation was requested. Maternal and infant serum samples were sent to a toxoplasmosis reference laboratory (Laboratory for Specialty Diagnostics, Palo Alto, CA, USA) for paired *T. gondii* antibody testing.

A repeat abdominal ultrasound on day 17 revealed hepatosplenomegaly and ascites. Paracentesis yielded 110 mL of clear yellow fluid with a lymphocytic predominance (white blood cell (WBC) count 359/mm^3^, lymphocytes 81%). Bacterial cultures of the peritoneal fluid were negative. Peritoneal fluid cytology was negative for trophozoites by pathologist review, and peritoneal fluid was sent to the reference laboratory for *T. gondii* PCR testing.

Cerebrospinal fluid (CSF) analysis showed WBC 44/mm^3^ (66% lymphocytes, 33% monocytes), red blood cell (RBC) 2/mm^3^, glucose 51 mg/dL, and protein 179 mg/dL. CSF cultures and universal bacterial PCR testing were negative, as was *T. gondii*-specific PCR (University of Washington, Seattle, WA, USA). A repeat head ultrasound was normal. The infant did not undergo a head computed tomography (CT) scan or brain magnetic resonance imaging (MRI) due to clinical instability. Early signs did not point to the central nervous system disease. When neurologic involvement was suspected, the infant was critically ill on mechanical ventilation and vasopressor. Transport for MRI or CT was too risky, and the priority was stabilization. Ophthalmologic examination showed no evidence of chorioretinitis.

#### 2.1.4. Clinical Course and Treatment

Over the following days, the infant developed liver failure characterized by direct hyperbilirubinemia, thrombocytopenia, coagulopathy, and ascites. A peritoneal drain was inserted to manage the persistent ascites. Enteral pyrimethamine, sulfadiazine, and leucovorin were considered but were contraindicated due to high risk of necrotizing enterocolitis with serum lactate of 10.7 mmol/L. Intravenous trimethoprim-sulfamethoxazole (IV TMP-SMX) and clindamycin were also considered but not started, as IV TMP-SMX volumes would worsen fluid overload and ascites, with a risk of respiratory failure.

Hemophagocytic lymphohistiocytosis (HLH) was considered based on elevated ferritin (5547 ng/mL; reference range < 620 ng/mL), high soluble interleukin-2 receptor (8350 U/mL; reference range < 1940 U/mL), thrombocytopenia (23,000/mm^3^), anemia (hematocrit 28.4%), and hypofibrinogenemia (103 mg/dL). Further laboratory investigation, including tests of natural killer (NK) cell function, X-linked inhibitor of apoptosis protein (XIAP) testing, and signaling lymphocyte activation molecule-associated protein (SAP), were within normal limits. As a result, the patient was not given a diagnosis of HLH or treated with HLH-directed therapy. Clinical rapid exome sequencing was sent to Baylor Genetics (Houston, TX, USA), a leading clinical diagnostic laboratory that specializes in next-generation sequencing-based testing for rare and undiagnosed genetic disorders, including rapid exome sequencing panels used to evaluate infants with suspected inborn errors of metabolism, immunodeficiencies, or other genetic causes of critical illness, including HLH.

On day 25, the reference laboratory reported that the infant’s serum *T. gondii* IgG titer was positive at 1:64 according to the Sabin-Feldman Dye Test (negative, <1:16), IgM was positive according to the immunosorbent agglutination assay (ISAGA) method, and the *T. gondii* PCR was positive in both blood and peritoneal fluid. Maternal testing for *T. gondii* antibodies revealed an IgG titer of 1:8000 with high avidity, IgM enzyme-linked immunosorbent assay (ELISA) negative at 1.1 (negative < 2.0), and IgA ELISA positive at 3.1 (negative < 2.1). The infant was diagnosed with congenital toxoplasmosis, and treatment with pyrimethamine, sulfadiazine, and folinic acid was initiated on day 25.

On day 26, the infant acutely decompensated. Management required multiple inotropes, mechanical ventilation, and inhaled nitric oxide. Alternative therapies for liver failure, such as liver transplantation, were considered but deemed not feasible given the infant’s critical illness and active infection. Despite maximal cardiopulmonary support, the infant’s condition continued to decline. He passed away on day 27 after compassionate extubation once further aggressive interventions were determined to be futile.

#### 2.1.5. Autopsy Findings

Gross post-mortem examination revealed generalized jaundice and icterus. The liver appeared mottled, and the lungs were grossly congested. The eyes were not examined. Representative sections from all major organs were processed according to standard anatomic pathology protocols and cut at 5 µm thickness, as described in Section 3.

**Figure 1:**
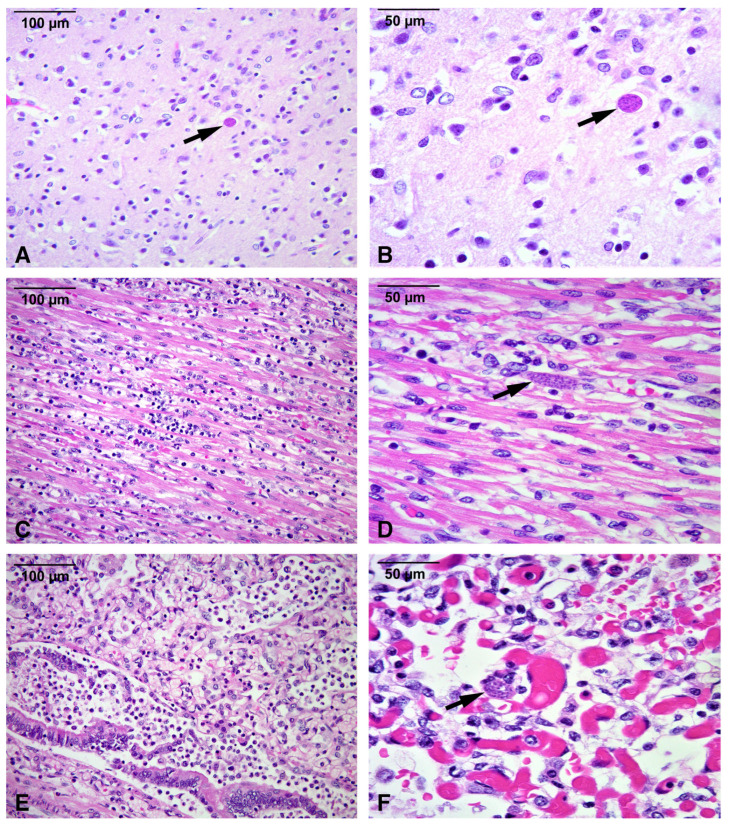
Histopathologic findings of disseminated congenital toxoplasmosis (hematoxylin and eosin). (**A**,**B**) Cerebral cortex showing increased mononuclear inflammatory infiltrates and a *T. gondii* cyst with bradyzoites (arrow) (**A**: 200×; **B**: 400×). (**C**,**D**) Myocardium with moderate inflammation and a *T. gondii* cyst with bradyzoites (arrow) (**C**: 200×; **D**: 400×). (**E**) Lung with severe mixed (predominantly acute) inflammation involving a bronchiole and adjacent alveoli (200×). (**F**) Congested pulmonary capillaries with a *T. gondii* cyst (arrow) (400×).

**Figure 2:**
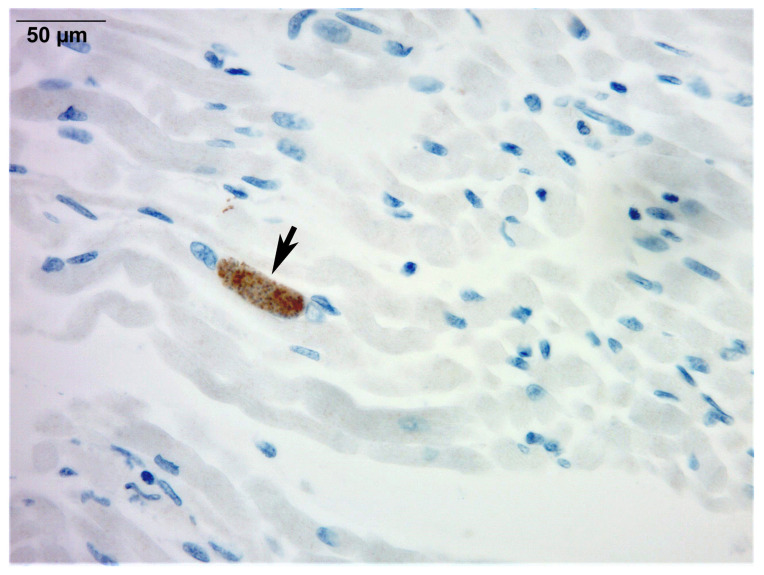
Skeletal muscle involvement in congenital toxoplasmosis (immunohistochemistry). Immunohistochemistry for *T. gondii* highlights a cyst containing bradyzoites (arrow) in skeletal muscle fibers (400×).

Microscopic examination demonstrated variable degrees of moderate to severe active and chronic inflammation involving the brain, heart, lungs, intestines, skeletal muscle, and urinary bladder. The liver showed extensive inflammation with submassive necrosis, while the spleen exhibited marked necrosis. Classic *T. gondii* cysts were scattered in multiple organs and were readily identified on routine hematoxylin and eosin staining (Figure 1). No histopathologic evidence of hemophagocytosis was observed, excluding HLH. Immunohistochemistry with a rabbit polyclonal anti-*T. gondii* IgG antibody (ab138698; Abcam, Cambridge, MA, USA) with standard antigen retrieval, peroxidase blocking, and 3,3′-diaminobenzidine (DAB) chromogen visualization confirmed the presence of the parasite (Figure 2), as detailed in Section 3. Postmortem PCR for *T. gondii* was carried out on formalin-fixed, paraffin-embedded liver tissue, using DNA extraction with standard deparaffinization and purification protocols, followed by amplification targeting the *B1* gene, as described in Section 3. The assay yielded positive results, further corroborating active *T. gondii* infection.

The inflammatory infiltrates consisted of mixed mononuclear and polymorphonuclear cells. Inflammation often exceeded the apparent parasite burden. Cysts were sometimes easier to find in less inflamed organs, such as heart and skeletal muscle. This pattern highlights the possibility of an exaggerated host immune response relative to parasite load. The cause of death was determined to be multiorgan failure secondary to active disseminated toxoplasmosis. The day‑by‑day clinical evolution, diagnostic evaluation, and therapeutic interventions from birth through autopsy are summarized in Table 1.

**Table 1:** Clinical timeline summarizing the infant’s presentation, diagnostic evaluation, and clinical progression. The table outlines key events from birth through post-mortem examination, including the early onset of thrombocytopenia and hepatitis, subsequent development of hepatosplenomegaly and progressive liver failure, diagnostic findings confirming congenital *T. gondii* infection, therapeutic decisions, and the evolution to multiorgan failure.

Postnatal Day	Key Events and Findings	Interventions/Outcomes
Birth (Day 0)	Male infant born at 38 weeks by emergency cesarean section. Birth weight 2680 g (7th percentile). Apgar 7/8. No hepatosplenomegaly. Labs: hypoglycemia, thrombocytopenia (16,000/mm^3^), elevated AST and direct bilirubin.	Non-invasive respiratory support. Platelet transfusions. IVIG × 3 for suspected NAIT (later excluded).
Day 6	*T. gondii* serologies drawn after IVIG: IgG elevated (37 IU/mL), IgM negative. Abdominal/head ultrasound: normal.	Supportive care continued.
Day 15	Fever, abdominal distension, leukocytosis.	Started nafcillin + gentamicin for presumed sepsis.
Day 16	Infectious disease consultation. Maternal/infant paired *T. gondii* serologies sent.	—
Day 17	Abdominal ultrasound: hepatosplenomegaly, ascites. Paracentesis: lymphocytic fluid. CSF: pleocytosis, elevated protein, PCR negative. Ophthalmology: no chorioretinitis.	Peritoneal fluid sent for *T. gondii* PCR.
Days 18–24	Progressive liver failure: direct hyperbilirubinemia, thrombocytopenia, coagulopathy, ascites. HLH considered (elevated ferritin, sIL-2R, cytopenias) but excluded by additional testing.	Peritoneal drain placed. Exome sequencing sent. Anti-*T. gondii* enteral therapy considered but contraindicated due to high lactate. Intravenous trimethoprim-sulfamethoxazole and clindamycin considered but not started due to volume concerns. Supportive care.
Day 25	Reference lab results: infant *T. gondii* IgG 1:64 (Sabin-Feldman), IgM positive (ISAGA), PCR positive in blood and peritoneal fluid. Maternal IgG 1:8000 (high avidity), IgM negative, IgA positive. Diagnosis: congenital toxoplasmosis.	Began pyrimethamine, sulfadiazine, folinic acid.
Day 26	Acute cardiopulmonary decompensation → mechanical ventilation, nitric oxide, inotropes.	Liver transplantation not feasible. Maximal support.
Day 27	Further deterioration despite intensive care.	Compassionate extubation. Death due to multiorgan failure secondary to disseminated toxoplasmosis.
Post-mortem	Autopsy: disseminated *T. gondii* infection with multi-organ inflammation and necrosis (liver, spleen, brain, heart, lungs, muscle, bladder). *T. gondii* cysts on hematoxylin and eosin. Immunohistochemistry and PCR confirmed infection.	Cause of death: multiorgan failure from disseminated congenital toxoplasmosis.

During his illness, the patient was enrolled in an Institutional Review Board (IRB)-approved study utilizing ultra-rapid genome sequencing (urGS) of blood mononuclear cells to identify genetic causes of liver failure, as previously described and as detailed in Section 3 [17,18]. Reads that did not align to the human reference genome (GRCh38) or to any other available human contigs in GenBank were reanalyzed for pathogen detection using kraken2 (v2.1.1) [19] and Bracken (v2.5) [20] within a custom, in-house metagenomics pipeline. Taxonomic classification was performed against a contamination-filtered EuPathDB46 kraken2 database of eukaryotic pathogens [21] downloaded from the Langmead laboratory AWS index repository (https://benlangmead.github.io/aws-indexes/k2 accessed on 27 December 2020); full parameter settings are provided in Section 3. This analysis identified 1679 read pairs aligning to the *T. gondii* ME49 reference genome, in contrast to more than 695 million reads that aligned to the human genome. No *T. gondii*-aligned reads were detected in concurrently analyzed non-human reads from the parental samples or in 463 unrelated adult and pediatric samples processed with the same pipeline, supporting a specific *T. gondii* infection in our index case. This metagenomic analysis has been described previously in abstract form [18].

### 2.2. Genomic Characterization of the Infecting Strain

To further investigate the genetic background of the infecting *T. gondii* strain TgHsUS2, using the approach detailed in Section 3, 3394 Illumina reads that did not align to the reference human assembly were remapped to the *T. gondii* ME49 (TgME49) reference sequence. Mapped reads were subsequently used to identify 2770 single nucleotide variants (SNVs), most of which were supported by a single read.

**Figure 3:**
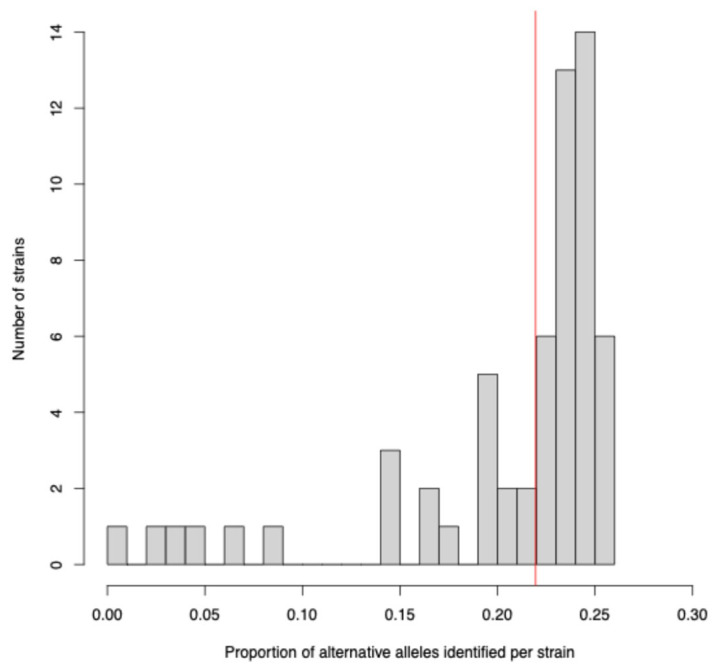
Histogram of SNV frequencies (mean number of SNVs per base pair) across 61 previously sequenced *T. gondii* strains. The vertical red line denotes the SNV frequency estimated for TgHsUS2. Gray bars represent the number of *T. gondii* strains with specific SNV frequencies.

To assess whether the identified SNVs resulted from sequencing errors, we compared the SNV frequency of TgHsUS2 against a set of previously reported polymorphic sites generated from 61 *T. gondii* reference strains (Figure 3) [22]. The result indicated that the SNV frequency in TgHsUS2 (0.22) was within the same range as that observed in other strains sequenced at a much higher depth (0.005–0.253), suggesting that most identified SNVs were likely authentic.

**Figure 4:**
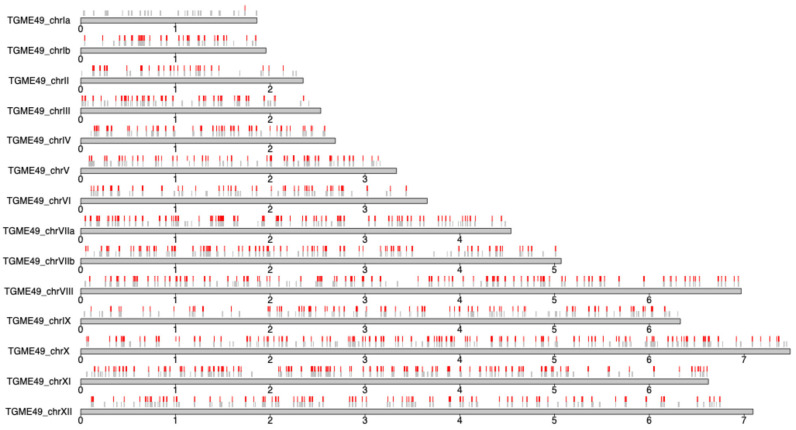
Genomic distribution of TgHsUS2 SNVs analyzed in this study. Gray and red vertical lines represent SNVs conserved or not between TgHsUS2 and TgME49, respectively. Gray horizontal lines represent chromosomes with chromosome names depicted on the left. Scales underneath chromosomes indicate chromosomal positions in megabases.

To refine the SNV dataset, all variants unique to TgHsUS2 and absent from the population of 61 *T. gondii* strains were excluded, yielding a dataset of 2295 high-confidence SNVs. In addition, 8159 sites conserved between TgHsUS2 and TgME49 but polymorphic in at least one of the 61 *T. gondii* strains, were incorporated generating a final dataset of 10,454 polymorphic sites. The genomic distribution of these SNVs was then plotted along the TgME49 chromosomes, revealing no obvious bias in their genomic distribution (Figure 4). Moreover, only one of the 193 TgHsUS2 sites on chromosome Ia was polymorphic with ME49, which is consistent with the high degree of conservation of this chromosome across *T. gondii* strains [23].

**Figure 5:**
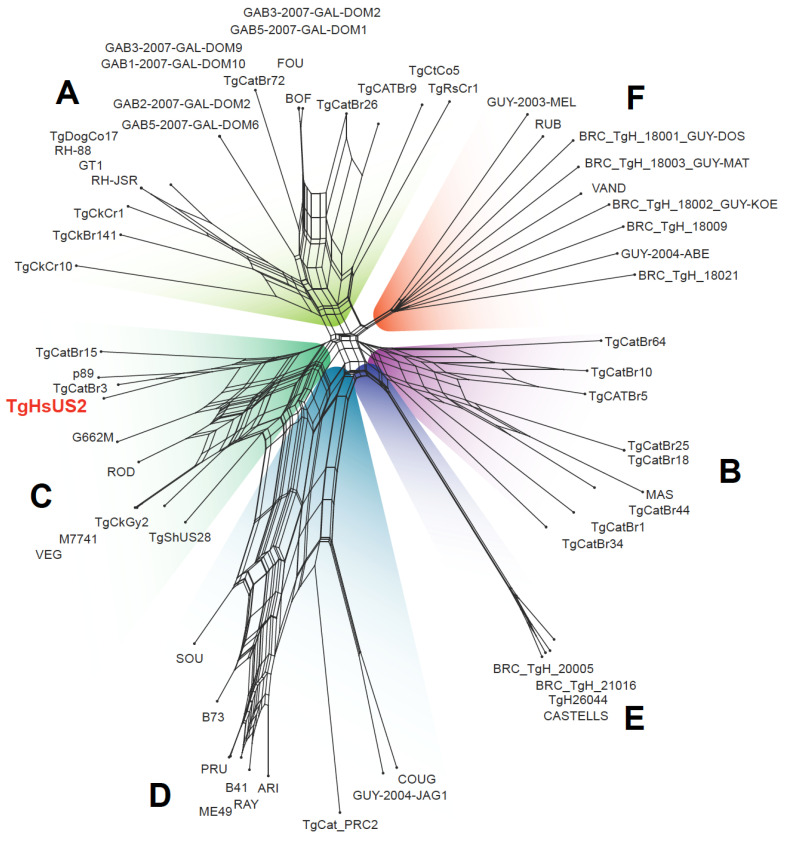
Phylogenetic network tree of 61 *T. gondii* reference strains and TgHsUS2. The names of the six known clades previously identified in the *T. gondii* population are indicated in bold capital letters (**A**–**F**). Strains belonging to the same clade share the same background color. The position of TgHsUS2 within clade (**C**) is highlighted in red.

A phylogenetic network tree was then constructed using the refined SNV dataset alongside the 61 reference strains, which span the six major *T. gondii* clades (A–F) (Figure 5). Network analysis showed that TgHsUS2 was closely related to strains P89 and TgCatBr3, both from clade C haplogroup 9. P89 was originally isolated from a pig in the US while TgCatBr3 corresponds to a Brazilian parasite isolated from a domestic cat [22].

**Figure 6:**
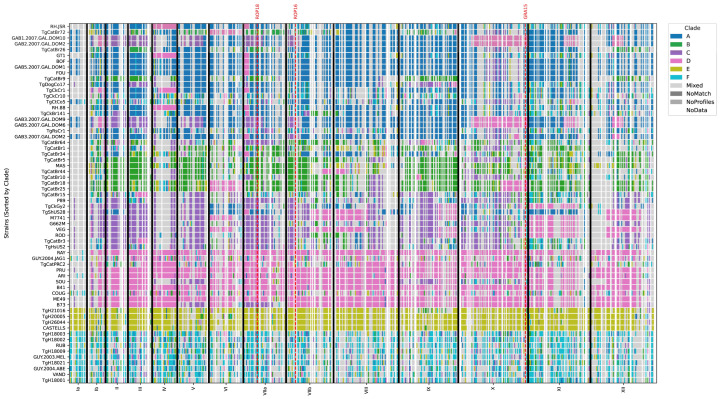
Chromosome painting of local admixture analysis based on shared SNV profiles within 100 kb bins across TgHsUS2 and the 61 *T. gondii* reference strains. Clade-specific SNV profiles are depicted in color: dark-blue (Clade A), green (Clade B), purple (Clade C), pink (Clade D), yellow (Clade E) and magenta (Clade F). Light gray bins denote SNV profiles that could not be assigned to one specific Clade (see Methods). Chromosome boundaries are indicated as black vertical lines. Red dashed lines denote chromosomal positions for virulence factors ROP18, ROP16 and GRA15. Strain names are indicated on the left and chromosome names are shown on the *x*-axis.

Previous studies have shown evidence of genetic crosses between *T. gondii* isolates from different clades [22,24]. To gain further insights into the degree of conservation between TgHsUS2 and its two closest relatives, P89 and TgCatBr3, we assessed the presence of local admixture patterns by comparing the similarity of SNV profiles within 100 Kbp bins across all 62 strains (Figure 6). This analysis confirmed that most of the TgHsUS2 genome is closest to P89 and TgCatBr3, except for the 3′ end of chromosome XII. In addition, TgHsUS2 chromosomes III and IX are closer to P89, while chromosomes VIIb, VIII, and X are more similar to TgCatBr3 (Figure S1).

Several *T. gondii* proteins secreted into the human host have been shown to play a role in the regulation of the immune response. Given the close similarity between TgHsUS2 and the two sequenced haplogroup 9 strains, we interrogated what was the most likely combinations of allelic variants encoded by TgHsUS2 for three virulence factors that are relevant to human infections, Rop18, Rop16, and Gra15 [25,26,27].

**Figure 7:**
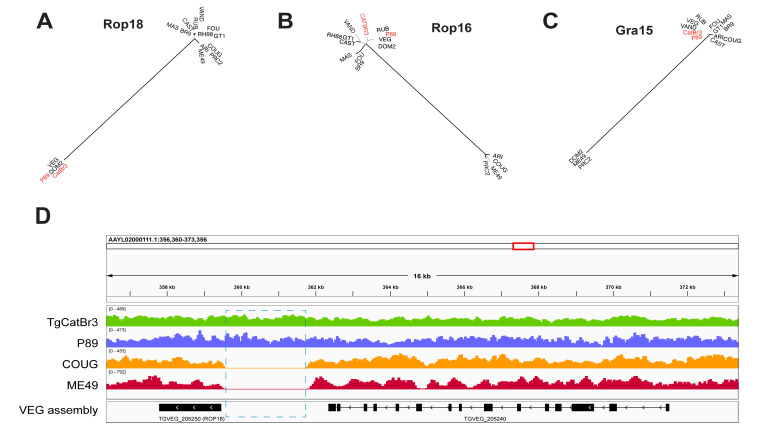
Phylogenetic analysis of three *T. gondii* virulence factors—Rop18 (**A**,**D**), Rop16 (**B**), and Gra15 (**C**)—among 15 *T. gondii* reference strains plus TgCatBr3. Black lines denote bootstrap values above 0.75. The positions of P89 and CatBr3, the two strains phylogenetically closest to TgHsUS2, are highlighted in red. Panel (**D**) depicts the read coverage along the *ROP18* locus of the type III VEG strain after mapping Illumina whole-genome sequencing reads from strains TgCatBr3, P89, COUG, and ME49 to the VEG genome assembly. The dashed rectangle indicates the region spanning the type III insertion present at the *ROP18* promoter of VEG, P89, and TgCatBr3, and absent in ME49 and COUG. Structural annotations of *ROP18* and a neighboring gene are shown at the bottom. The scale at the top of panel (**D**) indicates chromosomal positions on VEG chromosome TGVEG_chrVIIa.

Based on the SNV profile analysis, ROP18^TgHsUS2^ gene is encoded within a set of Clade C SNV profiles on chromosome VIIa (Purple block, Figure 6 and Figure S1). Multiple sequence alignment of the Rop18 protein encoded by the reference *T. gondii* strains of the 15 haplogroups described in [22] plus TgCatBr3 showed that both, Rop18^P89^ and Rop18^TgCatBr3^ proteins, are closely related to Rop18^VEG^, an avirulent, non-expressed type III allelic variant (Figure 7A). Both strains also carry the same insertion as the VEG strain in the promoter region of ROP18 (Figure 7D).

By phylogenetic analysis, Rop16 proteins can be sorted into two main groups, one encoded by strains belonging to Clade D and another encoded by strains from the other five Clades, including TgCatBr3 and P89 (Figure 7B). In TgHsUS2, the SNV profiles surrounding the ROP16 locus match those of P89 and TgCatBr3 (Figure 6 and Figure S1) and therefore, it is expected that ROP16^TgHsUS2^ allele is closely related to either ROP16^P89^ or ROP16^CatBr3^. ROP16^P89^ protein is identical to the type III isoform encoded by the VEG strain, while ROP16^CatBr3^ protein carries a single S458K mutation within the protein kinase catalytic domain.

As with Rop16, Gra15 proteins can be grouped into two main types, one composed of Clade D-Gra15 proteins and the second encompassing the remaining five clades (Figure 7C). Both, Gra15^CatBr3^ and Gra15^P89^, are phylogenetically related with the second group.

## 3. Methods

Autopsy tissue from major organs was processed using standard anatomic pathology protocols. Representative samples were fixed, processed, paraffin-embedded, and sectioned at 5 µm. Sections were stained with hematoxylin and eosin for routine histologic evaluation. For detection of *T. gondii*, immunohistochemistry was performed on formalin-fixed, paraffin-embedded sections using a rabbit polyclonal anti-*T. gondii* IgG antibody (ab138698; Abcam, Cambridge, MA), with standard antigen retrieval, endogenous peroxidase blocking, and DAB chromogen visualization. Postmortem PCR for *T. gondii* was carried out on formalin-fixed, paraffin-embedded liver tissue using DNA extraction with standard deparaffinization and purification procedures, followed by amplification targeting the *T. gondii* B1 gene.

Genome sequencing was carried out on Illumina NovaSeq sequencers (BioProject PRJNA1283239). Reads were first aligned to the human reference genome (GRCh38), and reads that did not map were extracted and used for metagenomic taxonomic classification and downstream parasite genomic analyses. For metagenomic pathogen detection, unmapped reads were analyzed with kraken2 (v2.1.1) [19] using a curated EuPathDB46 kraken2 database of eukaryotic pathogens [21] in which contaminant sequences had been removed (database downloaded 27 December 2020 from the Langmead laboratory AWS index repository). Kraken2 was run in paired-end mode with the options --paired, --use-names, --db, --threads 24, --confidence 0.75, and --report. Species-level abundance estimates were then obtained with Bracken (v2.5) [20] using the same database and parameters -d, -i, -o, and -t 10. For strain-level genomic characterization, the same unmapped reads were subsequently mapped to the *T. gondii* ME49 reference genome (ToxoDB-8.0) with bowtie2 (v2.5.4) using end-to-end mode. A total of 2989 non-duplicated reads mapped to ME49, spanning 394,832 bp (0.63%) of the ME49 assembly. Of these, 345,186 bp were supported by a single read and 49,646 bp were spanned by two to four reads. The resulting BAM file was sorted by genomic coordinates and processed to generate a pileup file with samtools mpileup (v1.20). Afterwards, SNVs were identified with samtools call by setting ploidy to 1 and --multiallelic-caller and then compared against a dataset of SNVs (REF_SNVs) previously identified across 61 *T. gondii* reference strains [22]. All conserved and SNV alleles from the TgHsUS2 strain whose genomic positions overlapped with variants from the REF_SNV dataset were concatenated together to build a DNA sequence in fasta format. Similar sequences were generated using equivalent alleles derived from each of the 61 *T. gondii* reference strains. Next, fasta DNA sequences were imported into SplitTree (v6.3.34) to build a phylogenetic network tree. The distribution of SNVs along chromosomes was visualized with the R package karyoploteR (v1.32.0) using an R (v4.4.2) custom script.

Chromosome paintings were generated using an in-house python script. Briefly, the reference *T. gondii* genome sequence was split into 100 kb-bins. Next, we identified all SNVs falling within each 100 kb bin and then, for each clade (A-F), we determined the representative allele profile based on the most frequent allele at each SNV position among strains belonging to the same clade. Next, we compared each strain’s allele pattern within each bin to the representative clade profiles and then assigned the bin to the clade with the highest percent identity, flagging profiles as “Mixed” in case of ties. Finally, strains were sorted by clade and bins were concatenated by position to plot the profiles along the genome and plotted using the python matplotlib library.

For phylogenetic analysis, protein sequences for Rop18, Rop16 and Gra15 for the 16 *T. gondii* reference assemblies from Lorenzi et al. [22], were fetched from ToxoDB (release 68). For TgCatBr3, Illumina paired-end sequencing reads were downloaded from SRA (SRR521971), trimmed with fastp (v0.24.0) to remove adapter sequences, mapped with bowtie2 (v2.5.4) to the P89 reference assembly (ToxoDB release 68) and then a TgCatBr3 assembly consensus sequence was generated from the resulting bam file with pilon (v1.24) using default settings. Rop18, Rsop16 and Gra15 protein sequences for TgCatBr3 where then reconstructed from generating tblastn-based alignments with the corresponding protein sequences from P89 using TgCatBr3 consensus assembly as subject. Thereafter, multiple sequence alignment and phylogenetic trees were performed with clustalw (v2.1) with BOOTSTRAP = 1000, OUTPUTTREE = phylip, and ALIGN parameters. Trees were plotted with UGENE software (v52.0) [28].

## 4. Discussion

We present a case of a critically ill infant suffering from disseminated congenital toxoplasmosis that progressed to fulminant liver failure. The infant initially displayed thrombocytopenia and mild liver dysfunction, followed by rapid deterioration with respiratory failure, severe coagulopathy, and ascites. Autopsy revealed severe disseminated congenital toxoplasmosis involving multiple organs. The severity and fatal outcome of this case highlight the critical importance of swift diagnosis, early initiation of appropriate therapy, and effective prevention strategies.

In our literature review spanning the last two decades, we identified only four reported instances of disseminated congenital toxoplasmosis leading to multi-organ failure (Table 2) [29,30,31,32]. These cases, together with ours, consistently manifested thrombocytopenia, often accompanied by coagulopathy and respiratory distress. Three of the four previously reported cases had abnormal findings involving either the brain or eyes, making our case, in which such abnormalities were not documented, an uncommon presentation.

This case was distinguished by predominant hepatic failure. The classic triad of congenital toxoplasmosis—chorioretinitis, intracranial calcifications, and hydrocephalus—was absent. While hepatic involvement has been described, progression to fulminant liver failure as the dominant clinical course is rarely described [15]. Moreover, the genomic characterization of the infecting strain, TgHsUS2, revealed type III-like allelic variants in key virulence genes, suggesting immune-modulatory properties distinct from the more common type II strains in Europe. Thus, this case not only expands the clinical spectrum of congenital toxoplasmosis beyond neurologic and ocular disease but also supports a potential contribution of strain diversity to atypical disease phenotypes.

The combination of abnormal liver function tests, thrombocytopenia, CSF pleocytosis, and ascites in the newborn period, together with positive IgG antibodies, generally constitutes an indication for empiric treatment while definitive diagnostic tests are pending [2]. In this case, multi-organ critical illness complicated decisions regarding empiric treatment, and diagnostic uncertainty in interpreting the initial negative IgM and CSF PCR results contributed to delayed initiation of therapy until confirmatory testing was available on day 25.

Serologic testing of the mother is particularly valuable in infants who have received transfusions or IVIG, as in our patient, because neonatal serologic results may be difficult to interpret [33]. IVIG may have influenced the initial negative IgM through passive antibodies or immunomodulatory effects [34,35]. Despite this potential confounder, reference testing (Sabin-Feldman Dye Test, ISAGA IgM, and PCR from blood and peritoneal fluid) established the diagnosis with high specificity. These assays are well validated for use in neonates with suspected congenital toxoplasmosis and represent the diagnostic standard in reference laboratories worldwide [33,36].

Maternal serologies revealed a high IgG titer with high avidity, consistent with past infection, together with a positive IgA. This mixed profile can occasionally be observed when a mother with latent infection experiences subclinical reactivation during pregnancy, or when IgA persists longer than expected after an earlier infection [37,38]. Although the precise timing of maternal infection could not be established, the elevated IgA raises the possibility of infection or reactivation during gestation, allowing transplacental transmission [37,38]. This illustrates the challenges of interpreting maternal serology in isolation and underscores the importance of confirmatory neonatal testing in such cases [33].

While the timing of IVIG administration limits early interpretation of the infant’s IgM results, and maternal serology suggested prior infection with possible recent activity, these factors do not alter the overall diagnostic certainty in this case. Instead, they emphasize how congenital toxoplasmosis can produce severe neonatal disease and how diagnostic complexity can delay treatment. Together, these observations reinforce the importance of gestational screening, which can enable prompt diagnosis and rapid initiation of therapy [39].

Table 2 summarizes our case alongside four previously reported cases of severe disseminated congenital toxoplasmosis, incorporating available parasite genotype data. Where parasite genotyping has been performed, the implicated strains have been heterogeneous. In the case reported by Elbez-Rubinstein et al., the infant was infected with an atypical South American-like genotype acquired after maternal consumption of imported raw horse meat; despite a septic-shock-like presentation with multiorgan involvement, the infant survived with early diagnosis and treatment [30]. In contrast, Kieffer et al. described a fatal disseminated congenital infection due to a canonical type II strain, with death on day 10 despite initiation of therapy on day 4 [32]. The other two disseminated neonatal cases did not include parasite typing but showed similarly fulminant systemic illness [29,31]. Our case expands this spectrum by documenting a clade C (haplogroup 9) strain with type III-like ROP18, ROP16, and GRA15 alleles associated with fatal liver failure and multiorgan disease. Together with cohort data from the NCCCTS, which demonstrated that NE-II serotypes are over-represented among infants with severe manifestations at birth compared with type II infections [14], these findings suggest that severe disseminated congenital toxoplasmosis can arise from diverse genotypes, with atypical and non-type II lineages—including TgHsUS2—prominent among the most severe presentations.

More broadly, the serotype and genotype of *T. gondii*, particularly atypical lineages, are considered risk factors for more severe clinical presentations [14,40,41]. An atypical genotype has been associated with a rapid increase in severe toxoplasmosis among immunocompetent adults in French Guiana [42]. In a large US cohort, the NCCCTS compared outcomes of congenital toxoplasmosis caused by type II versus non-exclusively serotype II (NE-II) and found an association between severe disease at birth and NE-II infection [14]. In contrast, a more recent US genotyping study did not identify a significant correlation between disease severity and genotype, but the authors acknowledged that missing data may have limited statistical power [43]. Although the conclusions of these two studies differ, both indicate that atypical serotypes account for a substantial proportion (50–60%) of congenital toxoplasmosis cases in the US [14,43], in contrast to France, where 90% of cases are caused by the type II strains [44]. Taken together, these data support the hypothesis that strain diversity in the US may contribute to clinical severity, in contrast to regions dominated by type II lineages.

These observations are consistent with broader global patterns. Worldwide, *T. gondii* strain distribution and virulence vary substantially, shaping disease burden and clinical outcomes. The estimated global human seroprevalence is about 25–36% [45]. In Europe, type II strains predominate, accounting for most human isolates and nearly all congenital cases in France [44], where they are typically associated with ocular or neurologic disease rather than fulminant disseminated illness. In contrast, South America harbors striking diversity of atypical and recombinant lineages [46,47], many of which are highly virulent and associated with severe ocular disease or life-threatening disseminated toxoplasmosis in immunocompetent adults, as illustrated by outbreaks in French Guiana [42]. North America shows a more mixed pattern, with approximately equal proportions of type II and atypical strains, and close to half of congenital cases attributable to non-type II genotypes [13,43]. In Asia, diversity appears more limited, with ToxoDB#9 (Chinese 1) most common, although admixture with South and North American lineages has been observed, suggesting complex evolutionary origins [48,49]. Collectively, these global patterns underscore that strain distribution is a critical determinant of disease severity and place the unusually severe presentation of our case within the broader epidemiology of *T. gondii*.

From a population-genetic standpoint, *T. gondii* isolates worldwide cluster into six major clades and at least fifteen haplogroups, reflecting repeated recombination events followed by clonal expansion of successful lineages [22,50]. Clade C encompasses the classical type III lineage (haplogroup 3) and the closely related haplogroup 9, which includes the reference strains P89 (pig, Iowa, USA) and TgCatBr3 (domestic cat, Brazil). These clade C strains share large conserved haplotype blocks, including a characteristic chromosome Ia and clusters of secreted pathogenesis determinants, consistent with recent admixture and selection for traits that enhance transmission and host adaptation in domestic cycles [22,50].

In the US and other parts of the Northern Hemisphere, clade C/type III-like parasites are widely represented in domestic and wild animal reservoirs. Surveys of pigs, lambs and retail meat have shown that type II and type III lineages, together with a highly prevalent ‘fourth clonal type’ (haplogroup 12), account for most viable *T. gondii* isolates recovered from food animals, indicating that a limited set of intercontinental lineages dominate the domestic cycle [51,52,53,54]. Transplacental infections and a spectrum of clonal and atypical genotypes have also been documented in white-tailed deer and other wildlife, highlighting frequent exchange between sylvatic and domestic cycles in North America [51]. Human genotyping studies likewise show that, in addition to type II and III strains, approximately 40–45% of infections are caused by atypical genotypes, several of which can be traced to North American animal reservoirs rather than to imported infections [43]. Within this framework, TgHsUS2 clusters within clade C/haplogroup 9 and is most closely related to P89, linking this fatal congenital infection to a lineage that circulates in U.S. food animals and domestic environments [22]. Together, these data suggest that the dominance of a restricted set of intercontinental clonal lineages in livestock, coupled with frequent mixing of domestic and sylvatic transmission cycles, are key drivers of the emergence and persistence of clade C strains in the US.

Using Illumina sequencing of DNA extracted from the patient’s blood sample, we identified TgHsUS2 as taxonomically related to strains TgCatBr3 and P89, both from haplogroup 9, Clade C. This clade also encompasses type III strains, which are avirulent in mice, partly due to an insertion in the promoter region of the major virulence factor ROP18 that prevents its expression [55]. In humans, this non-expressing allele has been reported to be negatively associated with severe ocular toxoplasmosis [26]. TgCatBr3 and P89 share the same haploblock spanning the ROP18 gene with TgHsUS2, and their Rop18 proteins are closely related to Rop18^VEG^. Sequence alignment further confirmed that these strains, like type III strains, carry the promoter insertion.

Phylogenetic analysis also revealed that P89 and TgCatBr3 Rop16 and Gra15 proteins are similar to the type III allelic variants. These proteins are known to modulate host immune response in a strain-dependent manner. Rop16-type I/III, but not type II, phosphorylates host STAT3 and STAT6, promoting early M2 macrophage polarization and reduced IL-12 secretion, favoring cyst formation [25]. In contrast, Gra15-type II, but not type I/III, activates NF-kB and induces a pro-inflammatory response [27]. Beyond NF-κB signaling, GRA15 directly increases parasite susceptibility to IFNγ-dependent, cell-autonomous immunity by recruiting TRAF2/6 to the parasitophorous vacuole membrane, which in human fibroblasts promotes p62/NDP52 and LC3B/GABARAP loading and lysosomal destruction of the vacuole; in murine fibroblasts, GRA15-mediated TRAF6 recruitment drives IRG/GBP loading and vacuole disruption [56]. Thus, TgHsUS2, like other type III-like strains, may blunt protective Th1 responses by reducing IL-12 production and skewing macrophages toward an anti-inflammatory state.

Altogether, the combined virulence gene profile suggests that TgHsUS2 likely elicited a type III-like immune response in our patient. Although type III strains are generally less virulent than type I strains in murine models, host susceptibility varies substantially across species and even among individuals of the same species, influenced by genetic factors such as inflammasome-related loci and ALOX12 variants in rat models [57]. Old World primates are relatively resistant to *T. gondii*, whereas New World primates exhibit marked susceptibility [58]. Similar inter-individual variability in humans is plausible given known genetic determinants of innate immune function [59,60]. Within this context, the specific allelic configuration of ROP18, ROP16, and GRA15 in TgHsUS2 may have limited effective early immune activation in this neonate. When combined with the immunologic immaturity of the newborn and the absence of timely maternal treatment, these factors likely contributed to the unusually fulminant and disseminated clinical course. While parasite genotype alone cannot fully account for disease severity, the genomic data provide important insight into the host–pathogen interactions underlying this fatal infection.

Several limitations must be acknowledged. First, this is a single-case report, and the generalizability of our conclusions is inherently limited. Second, our genomic characterization of TgHsUS2 relied on sequence alignment with reference strains and in silico inference of virulence-associated loci; we did not isolate viable parasites or perform functional assays (e.g., infection of host cells or animal models) to directly test the virulence or immune-modulatory effects of the predicted type III-like ROP18, ROP16, and GRA15 alleles. Additional in vitro and in vivo studies will be required to determine whether TgHsUS2 behaves like canonical type III strains or whether the combination of parasite genotype, host factors, and timing of infection produces a distinct virulence phenotype. Accordingly, our interpretations regarding strain virulence and host–pathogen interactions should be viewed as hypothesis-generating rather than definitive.

In rare cases, disseminated congenital toxoplasmosis can be the presentation manifestation of primary immunodeficiency [61]. Activated PI3-Kinase Delta Syndrome type II (APDS2), for example, was discovered in an infant with disseminated congenital toxoplasmosis and subsequently in the mother [61]. PI3-Kinase plays a pivotal role in regulating cellular activation, growth, and differentiation, and activating mutations in APDS2 lead to immune dysregulation. In addition, maternal immunosuppression due to medications [62] or HIV infection [63] may impair the immune response and result in congenital toxoplasmosis in the child. Notably, genome sequencing in our patient did not reveal variants suggestive of a congenital immunodeficiency.

In this case, we initiated the treatment regimen endorsed by the NCCCTS [13] only two days before the patient’s death. The standard postnatal treatment protocol for congenital toxoplasmosis consists of pyrimethamine, sulfadiazine, and folinic acid [2]. Treatment protocols for dosage and duration have been evaluated [13], and more recent observational studies examining long-term outcomes, all using a uniform regimen with follow-up from 1981 to the present in the US, have demonstrated sustained benefit [14,64,65]. Several cohort studies have shown improved outcomes in infants with congenital toxoplasmosis who received treatment compared with historical control cases [13,39,66,67]. Congenital toxoplasmosis is therefore both preventable and treatable [65]. This case illustrates the critical importance of early diagnosis and prompt initiation of therapy.

Prenatal screening and treatment of seroconverting pregnant women have been shown to reduce both the incidence and severity of congenital toxoplasmosis [67,68,69,70,71]. These interventions are cost-effective for healthcare systems even in low-prevalence countries such as Austria [72] and France [73]. Several European countries have implemented free national routine antenatal screening programs [67,74] and postnatal surveillance programs [75]. The impact of these strategies in mitigating complications of symptomatic congenital toxoplasmosis and improving newborn outcomes is substantial [66,67,68,76]. In France, adoption of regular monthly screening, amniotic fluid PCR, immediate pyrimethamine-sulfadiazine treatment upon diagnosis, and follow-up postnatal therapy in 1995 led to a decrease in the rate of symptomatic congenital toxoplasmosis from 11% to 4% [67]. A 2007 meta-analysis of European cohort data provided only limited evidence that antenatal treatment reduces the risk of congenital toxoplasmosis [77], but this analysis may have underestimated treatment efficacy, as it did not include cohorts with the highest disease burden from North and South America and combined heterogeneous management strategies [2]. In contrast, when uniform approaches were implemented in France, substantial benefits and cost-effectiveness were demonstrated. Given the markedly elevated maternal IgG titer in our case, a prenatal screening program would likely have enabled earlier maternal diagnosis and treatment, potentially improving the infant’s outcome or even preventing disease.

Over the past five years, additional evidence has reinforced the view that congenital toxoplasmosis is both preventable and treatable when maternal infection is detected early. A recent cohort from southern Brazil showed that children born to untreated women with acute gestational toxoplasmosis had an approximately 6.5-fold higher risk of congenital infection, with a vertical transmission rate of 50% compared with 8.3% among those whose mothers received standard spiramycin or pyrimethamine–sulfadiazine–folinic acid therapy [78]. Similarly, a large national registry study from Spain (REIV-TOXO, 2015–2022) reported that prenatal treatment was associated with milder clinical manifestations at birth and during follow-up, supporting the continued value of organized prenatal screening programs [79]. These findings are consistent with a recent systematic review and meta-analysis including 56 cohort studies and more than 11,000 pregnant women, which demonstrated that gestational treatment reduced the risk of fetal infection (risk ratio ~0.34) and of clinical sequelae in infected neonates (risk ratio ~0.30), with triple therapy showing the most consistent benefit [39]. In parallel, screening strategies have advanced. An immunochromatographic IgG/IgM test (Toxoplasma immunochromatographic test [ICT]) has shown very high sensitivity (99.3%) and specificity (100%) compared with automated platforms, while requiring no specialized equipment, making it suitable for routine prenatal screening and use in resource-limited settings [70]. Building on this, a recent multicenter trial validated a monthly gestational screening paradigm using point-of-care ICT, which accurately identified acute maternal infections, reduced false-positive IgM results, and facilitated rapid initiation of treatment to prevent congenital toxoplasmosis [71]. Our case, characterized by predominant hepatic failure, fulminant disseminated disease, and very late maternal and neonatal diagnosis, underscores the need to incorporate such modern screening and treatment strategies into routine antenatal care.

In conclusion, this case of severe disseminated congenital toxoplasmosis with progressive liver failure illustrates how delayed diagnosis and treatment, combined with an immature neonatal immune system and a type III-like parasite genotype, can culminate in a fatal outcome. Given the poor prognosis reported for disseminated congenital toxoplasmosis, even with timely intervention, systematic antenatal screening and prompt treatment of maternal infection are likely to provide substantial benefits and should be strongly considered as part of comprehensive maternal–child health programs.

## Figures and Tables

**Table 2:** Clinical characteristics and *T. gondii* strain genotypes of our case and published cases of severe disseminated congenital toxoplasmosis in the literature. This table summarizes the clinical features, diagnostic testing, maternal serologic profiles, *T. gondii* strain genotypes when reported, presenting symptoms, and outcomes of our case alongside four previously reported cases of severe disseminated congenital toxoplasmosis.

Patient	Age at Diagnosis	Diagnostic Testing of the Infant	Maternal Serologies	Clinical Presentation	Outcome	Year, Source	*T. gondii* Strain Genotype (if Reported)
1	Day 2	IgM+	IgG-, IgM-	Thrombocytopenia, coagulopathy, liver failure, respiratory failure, pulmonary hypertension	Survived	2004 [29]	Not reported
2	Day 8	IgM+, blood PCR+	IgG+, IgM-	Thrombocytopenia, hepatosplenomegaly, respiratory distress, chorioretinitis	Survived	2009 [30]	Atypical, South American-like microsatellite genotype (non-type II), isolated from newborn blood
3	Day 6	IgM+	IgG+, IgM+	Thrombocytopenia, coagulopathy, transaminitis, cardiac arrest, respiratory failure, pulmonary hemorrhage, pulmonary hypertension, chorioretinitis, intracranial calcification	Survived	2010 [31]	Not reported
4	Day 3	IgM+, PCR+ (blood, tracheal aspirate, urine, ascites)	IgG+, IgM+	Thrombocytopenia, coagulopathy, respiratory failure, pulmonary hypertension, hepatosplenomegaly, ascites, macular bleeding and edema	Death on day 10	2011 [32]	Type II strain on genotyping of infant samples
5	Day 25	IgM+, blood PCR+	IgG+, IgM-	Thrombocytopenia, liver failure, ascites, respiratory distress	Death on day 27	2020, present case	Clade C (haplogroup 9) isolate TgHsUS2, closely related to P89/TgCatBr3, with type III-like ROP18/ROP16/GRA15 alleles

## Data Availability

The original data presented in the study are openly available in Zenodo at https://doi.org/10.5281/zenodo.15723402. Code used for the taxonomic characterization of TgHsUS2 is available in Zenodo at https://doi.org/10.5281/zenodo.15757875.

## Supplementary Materials

The following supporting information can be downloaded at: https://www.mdpi.com/article/10.3390/microorganisms13122865/s1. Figure S1: Haplogroup assignments in 100‑kb bins across each *Toxoplasma gondii* ME49 chromosome. Each panel shows local ancestry (“chromosome painting”) for non‑overlapping 100‑kb bins along a single TgME49 chromosome (Ia, Ib, II–XII). Rows correspond to TgHsUS2 and the 61 reference *T. gondii* strains, ordered by clade on the y‑axis; columns represent genomic position in base pairs along the x‑axis. For each bin, color indicates the clade whose consensus SNV profile best matches that bin: dark blue, clade A; green, clade B; purple, clade C; pink, clade D; yellow, clade E; magenta, clade F. Gray shades denote bins classified as Mixed (equally similar to ≥2 clades), NoMatch (no clade meets the similarity threshold), or NoProfiles (insufficient data to define a clade consensus), and white indicates NoData for that strain/bin. Red dashed vertical lines mark the chromosomal positions of virulence loci ROP18 (chromosome VIIa), ROP16 (chromosome VIIb), and GRA15 (chromosome X). This figure provides a chromosome‑by‑chromosome view of the local admixture analysis summarized in the main‑text chromosome painting plot.

## Abbreviations

The following abbreviations are used in this manuscript:

HLH	Hemophagocytic lymphohistiocytosis
IVIG	Intravenous immunoglobulin
NE-II	Non-exclusively serotype II
SNV	Single-nucleotide variant
urGS	Ultra-rapid genome sequencing
