# Supplementary material for: Fatal Congenital Toxoplasmosis with Progressive Liver Failure and Genomic Characterization of a Novel Isolate from the United States
Source: Microorganisms. 2025 Dec 17;13(12):2865. doi: 10.3390/microorganisms13122865 (PMC12736225; doi:10.3390/microorganisms13122865)
Supplement: Supplementary file 1 [file microorganisms-13-02865-s001.zip › microorganisms-4031638-supplementary.pdf]

Haplogroup Assignments across TGME49_chrIa Bins (100kb)

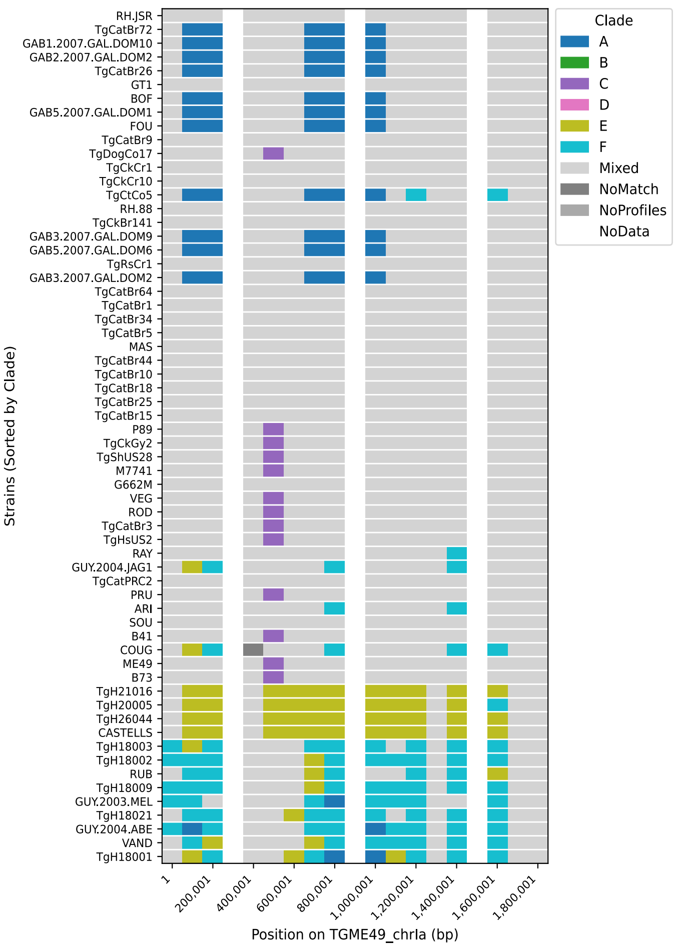

Haplogroup Assignments across TGME49_chrIb Bins (100kb)

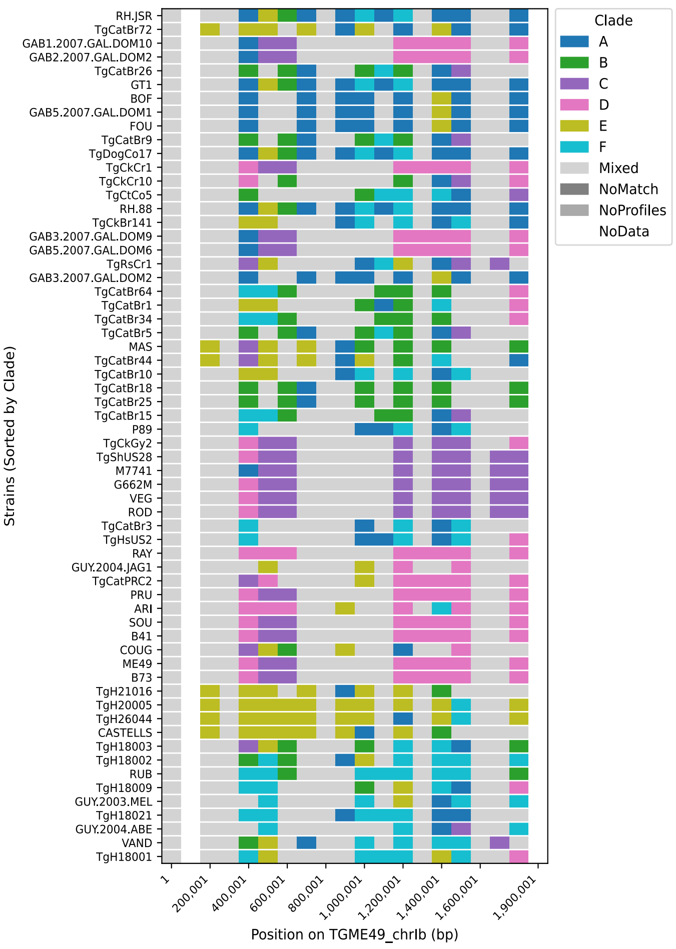

Haplogroup Assignments across TGME49_chrII Bins (100kb)

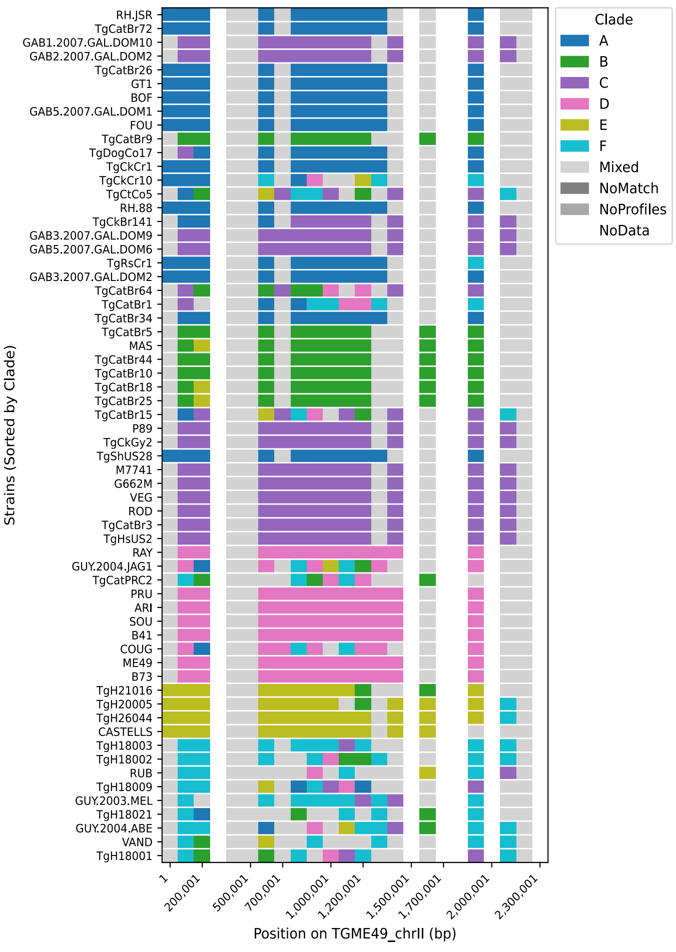

Haplogroup Assignments across TGME49_chrIII Bins (100kb)

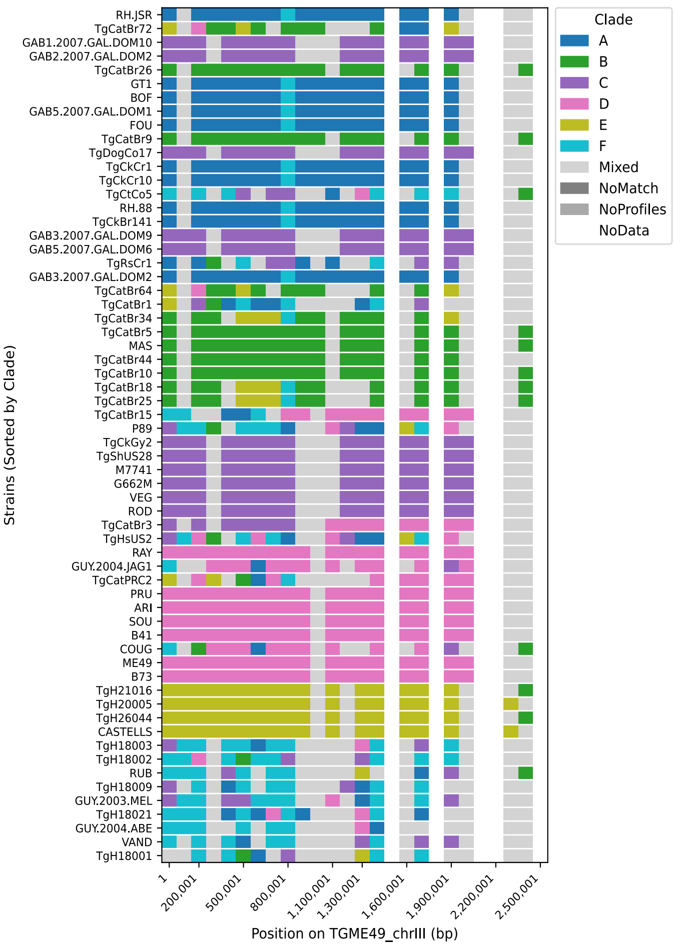

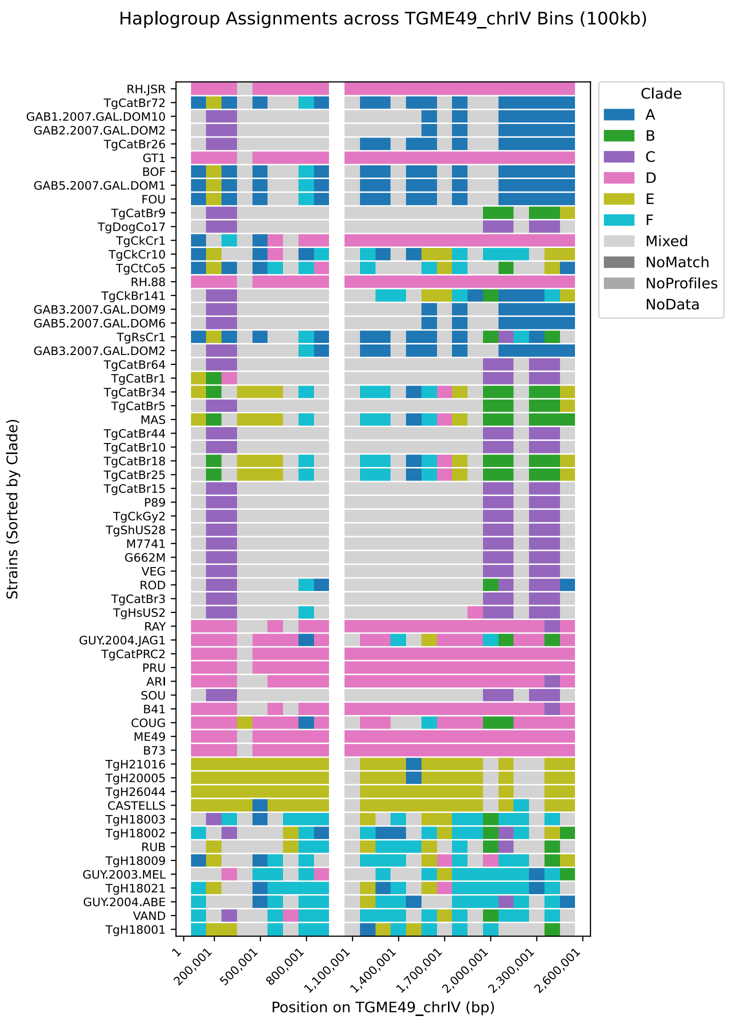
Haplogroup Assignments across TGME49_chrIV Bins (100kb)
Strains (Sorted by Clade)
RH.JSR
TgCatBr72
GAB1.2007.GAL.DOM10
GAB2.2007.GAL.DOM2
TgCatBr26
GT1
BOF
GAB5.2007.GAL.DOM1
FOU
TgCatBr9
TgDogCo17
TgCkCr1
TgCkCr10
TgCtCo5
RH.88
TgCkBr141
GAB3.2007.GAL.DOM9
GAB5.2007.GAL.DOM6
TgRsCr1
GAB3.2007.GAL.DOM2
TgCatBr64
TgCatBr1
TgCatBr34
TgCatBr5
MAS
TgCatBr44
TgCatBr10
TgCatBr18
TgCatBr25
TgCatBr15
P89
TgCkGy2
TgShUS28
M7741
G662M
VEG
ROD
TgCatBr3
TgHsUS2
RAY
GUY.2004.JAG1
TgCatPRC2
PRU
ARI
SOU
B41
COUG
ME49
B73
TgH21016
TgH20005
TgH26044
CASTELLS
TgH18003
TgH18002
RUB
TgH18009
GUY.2003.MEL
TgH18021
GUY.2004.ABE
VAND
TgH18001
1
200,001
500,001
800,001
1,100,001
1,400,001
1,700,001
2,000,001
2,300,001
2,600,001
Position on TGME49_chrIV (bp)
Clade
A
B
C
D
E
F
Mixed
NoMatch
NoProfiles
NoData

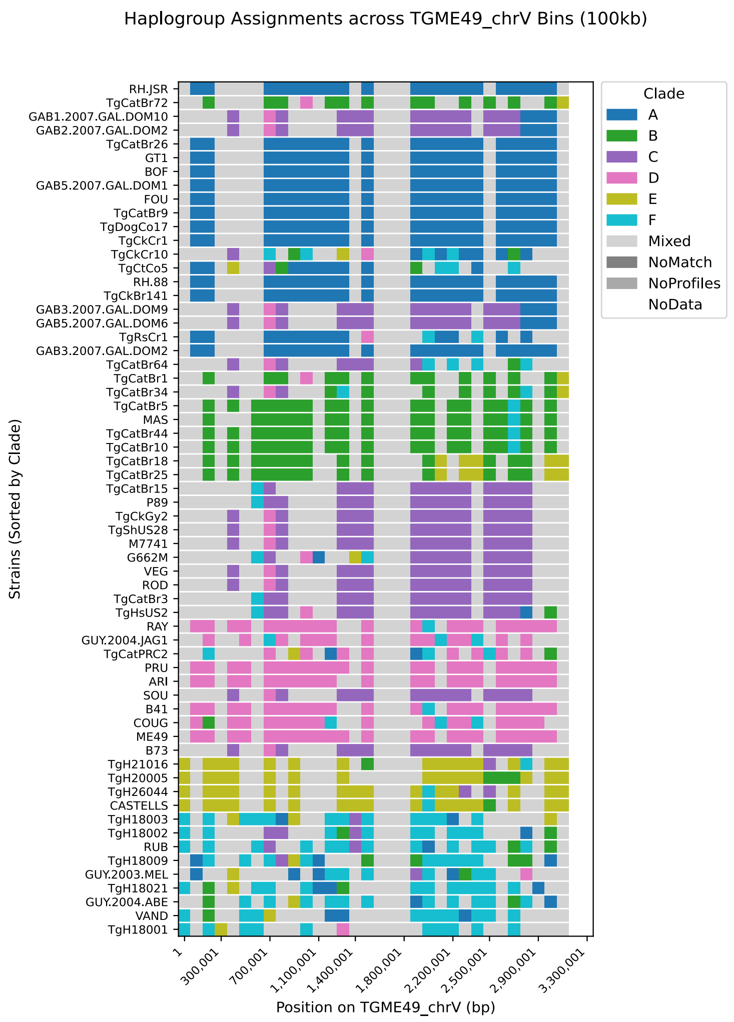
Haplogroup Assignments across TGME49_chrV Bins (100kb)
Strains (Sorted by Clade)
RH.JSR
TgCatBr72
GAB1.2007.GAL.DOM10
GAB2.2007.GAL.DOM2
TgCatBr26
GT1
BOF
GAB5.2007.GAL.DOM1
FOU
TgCatBr9
TgDogCo17
TgCkCr1
TgCkCr10
TgCtCo5
RH.88
TgCkBr141
GAB3.2007.GAL.DOM9
GAB5.2007.GAL.DOM6
TgRsCr1
GAB3.2007.GAL.DOM2
TgCatBr64
TgCatBr1
TgCatBr34
TgCatBr5
MAS
TgCatBr44
TgCatBr10
TgCatBr18
TgCatBr25
TgCatBr15
P89
TgCkGy2
TgShUS28
M7741
G662M
VEG
ROD
TgCatBr3
TgHsUS2
RAY
GUY.2004.JAG1
TgCatPRC2
PRU
ARI
SOU
B41
COUG
ME49
B73
TgH21016
TgH20005
TgH26044
CASTELLS
TgH18003
TgH18002
RUB
TgH18009
GUY.2003.MEL
TgH18021
GUY.2004.ABE
VAND
TgH18001
1
300,001
700,001
1,100,001
1,400,001
1,800,001
2,200,001
2,500,001
2,900,001
3,300,001
Position on TGME49_chrV (bp)
Clade
A
B
C
D
E
F
Mixed
NoMatch
NoProfiles
NoData

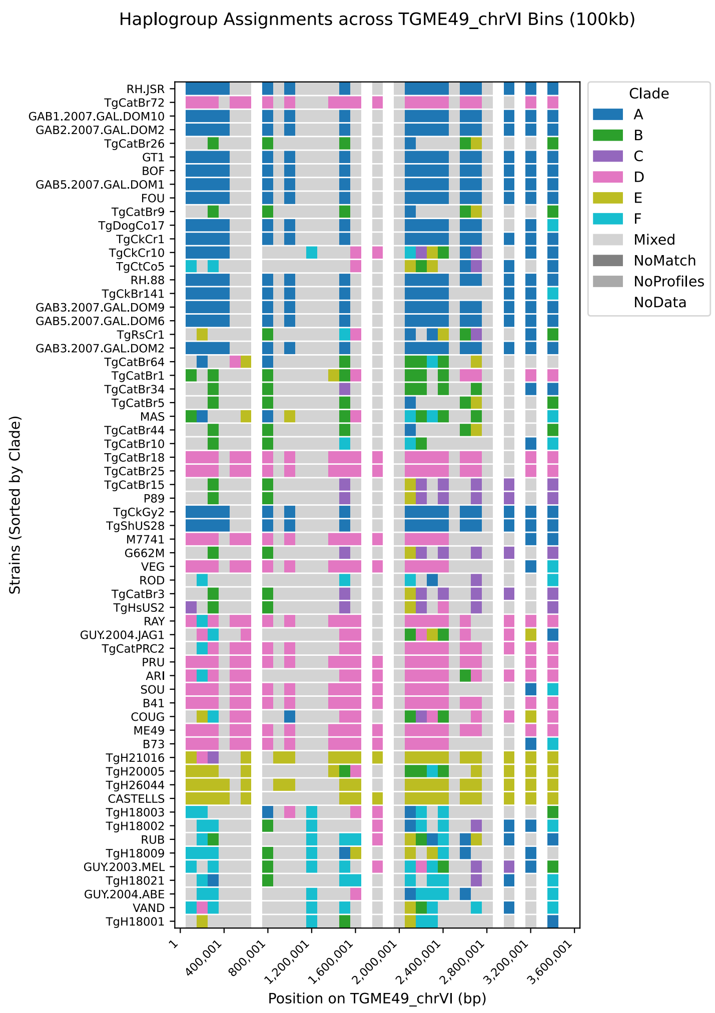
Haplogroup Assignments across TGME49_chrVI Bins (100kb)
Strains (Sorted by Clade)
RH.JSR
TgCatBr72
GAB1.2007.GAL.DOM10
GAB2.2007.GAL.DOM2
TgCatBr26
GT1
BOF
GAB5.2007.GAL.DOM1
FOU
TgCatBr9
TgDogCo17
TgCkCr1
TgCkCr10
TgCtCo5
RH.88
TgCkBr141
GAB3.2007.GAL.DOM9
GAB5.2007.GAL.DOM6
TgRsCr1
GAB3.2007.GAL.DOM2
TgCatBr64
TgCatBr1
TgCatBr34
TgCatBr5
MAS
TgCatBr44
TgCatBr10
TgCatBr18
TgCatBr25
TgCatBr15
P89
TgCkGy2
TgShUS28
M7741
G662M
VEG
ROD
TgCatBr3
TgHsUS2
RAY
GUY.2004.JAG1
TgCatPRC2
PRU
ARI
SOU
B41
COUG
ME49
B73
TgH21016
TgH20005
TgH26044
CASTELLS
TgH18003
TgH18002
RUB
TgH18009
GUY.2003.MEL
TgH18021
GUY.2004.ABE
VAND
TgH18001
1
400,001
800,001
1,200,001
1,600,001
2,000,001
2,400,001
2,800,001
3,200,001
3,600,001
Position on TGME49_chrVI (bp)
Clade
A
B
C
D
E
F
Mixed
NoMatch
NoProfiles
NoData

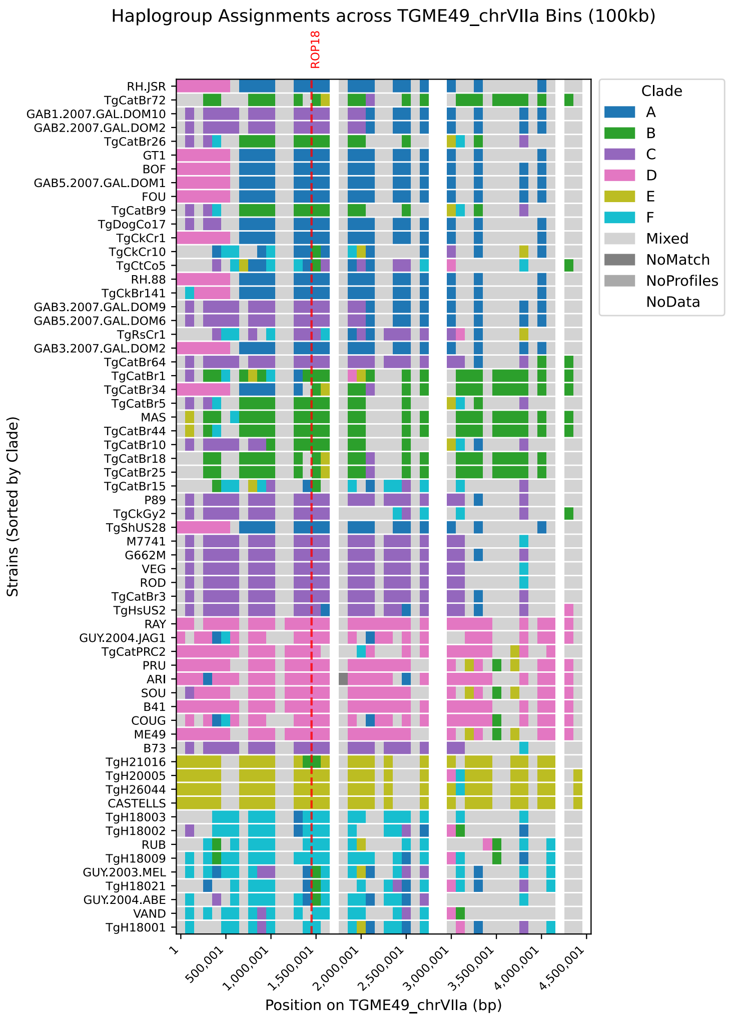
Haplogroup Assignments across TGME49_chrVIIa Bins (100kb)
ROP18
Strains (Sorted by Clade)
RH.JSR
TgCatBr72
GAB1.2007.GAL.DOM10
GAB2.2007.GAL.DOM2
TgCatBr26
GT1
BOF
GAB5.2007.GAL.DOM1
FOU
TgCatBr9
TgDogCo17
TgCkCr1
TgCkCr10
TgCtCo5
RH.88
TgCkBr141
GAB3.2007.GAL.DOM9
GAB5.2007.GAL.DOM6
TgRsCr1
GAB3.2007.GAL.DOM2
TgCatBr64
TgCatBr1
TgCatBr34
TgCatBr5
MAS
TgCatBr44
TgCatBr10
TgCatBr18
TgCatBr25
TgCatBr15
P89
TgCkGy2
TgShUS28
M7741
G662M
VEG
ROD
TgCatBr3
TgHsUS2
RAY
GUY.2004.JAG1
TgCatPRC2
PRU
ARI
SOU
B41
COUG
ME49
B73
TgH21016
TgH20005
TgH26044
CASTELLS
TgH18003
TgH18002
RUB
TgH18009
GUY.2003.MEL
TgH18021
GUY.2004.ABE
VAND
TgH18001
1
500,001
1,000,001
1,500,001
2,000,001
2,500,001
3,000,001
3,500,001
4,000,001
4,500,001
Position on TGME49_chrVIIa (bp)
Clade
A
B
C
D
E
F
Mixed
NoMatch
NoProfiles
NoData

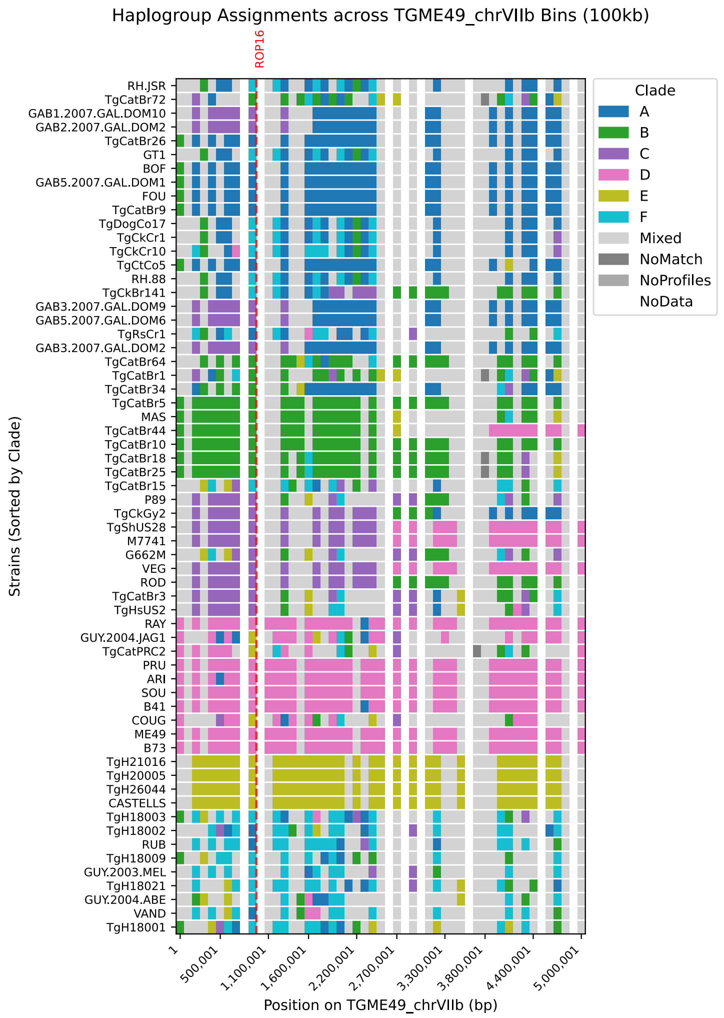
Haplogroup Assignments across TGME49_chrVIIb Bins (100kb)
ROP16
RH.JSR
TgCatBr72
GAB1.2007.GAL.DOM10
GAB2.2007.GAL.DOM2
TgCatBr26
GT1
BOF
GAB5.2007.GAL.DOM1
FOU
TgCatBr9
TgDogCo17
TgCkCr1
TgCkCr10
TgCtCo5
RH.88
TgCkBr141
GAB3.2007.GAL.DOM9
GAB5.2007.GAL.DOM6
TgRsCr1
GAB3.2007.GAL.DOM2
TgCatBr64
TgCatBr1
TgCatBr34
TgCatBr5
MAS
TgCatBr44
TgCatBr10
TgCatBr18
TgCatBr25
TgCatBr15
P89
TgCkGy2
TgShUS28
M7741
G662M
VEG
ROD
TgCatBr3
TgHsUS2
RAY
GUY.2004.JAG1
TgCatPRC2
PRU
ARI
SOU
B41
COUG
ME49
B73
TgH21016
TgH20005
TgH26044
CASTELLS
TgH18003
TgH18002
RUB
TgH18009
GUY.2003.MEL
TgH18021
GUY.2004.ABE
VAND
TgH18001
Strains (Sorted by Clade)
1
500,001
1,100,001
1,600,001
2,200,001
2,700,001
3,300,001
3,800,001
4,400,001
5,000,001
Position on TGME49_chrVIIb (bp)
Clade
A
B
C
D
E
F
Mixed
NoMatch
NoProfiles
NoData

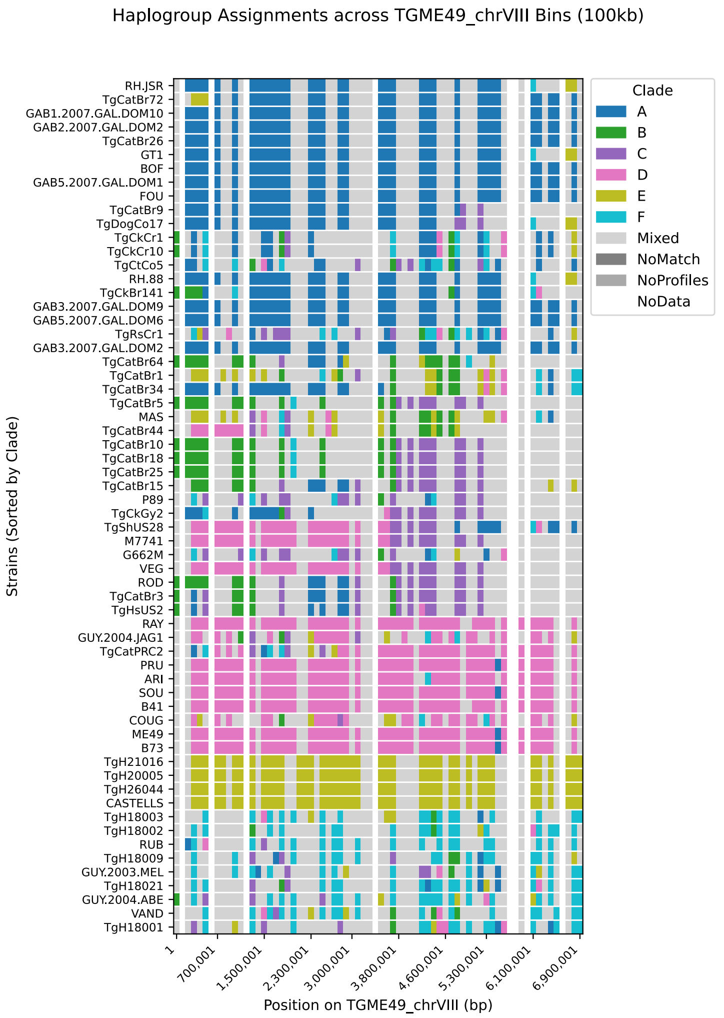
Haplogroup Assignments across TGME49_chrVIII Bins (100kb)
RH.JSR
TgCatBr72
GAB1.2007.GAL.DOM10
GAB2.2007.GAL.DOM2
TgCatBr26
GT1
BOF
GAB5.2007.GAL.DOM1
FOU
TgCatBr9
TgDogCo17
TgCkCr1
TgCkCr10
TgCtCo5
RH.88
TgCkBr141
GAB3.2007.GAL.DOM9
GAB5.2007.GAL.DOM6
TgRsCr1
GAB3.2007.GAL.DOM2
TgCatBr64
TgCatBr1
TgCatBr34
TgCatBr5
MAS
TgCatBr44
TgCatBr10
TgCatBr18
TgCatBr25
TgCatBr15
P89
TgCkGy2
TgShUS28
M7741
G662M
VEG
ROD
TgCatBr3
TgHsUS2
RAY
GUY.2004.JAG1
TgCatPRC2
PRU
ARI
SOU
B41
COUG
ME49
B73
TgH21016
TgH20005
TgH26044
CASTELLS
TgH18003
TgH18002
RUB
TgH18009
GUY.2003.MEL
TgH18021
GUY.2004.ABE
VAND
TgH18001
Strains (Sorted by Clade)
1
700,001
1,500,001
2,300,001
3,000,001
3,800,001
4,600,001
5,300,001
6,100,001
6,900,001
Position on TGME49_chrVIII (bp)
Clade
A
B
C
D
E
F
Mixed
NoMatch
NoProfiles
NoData

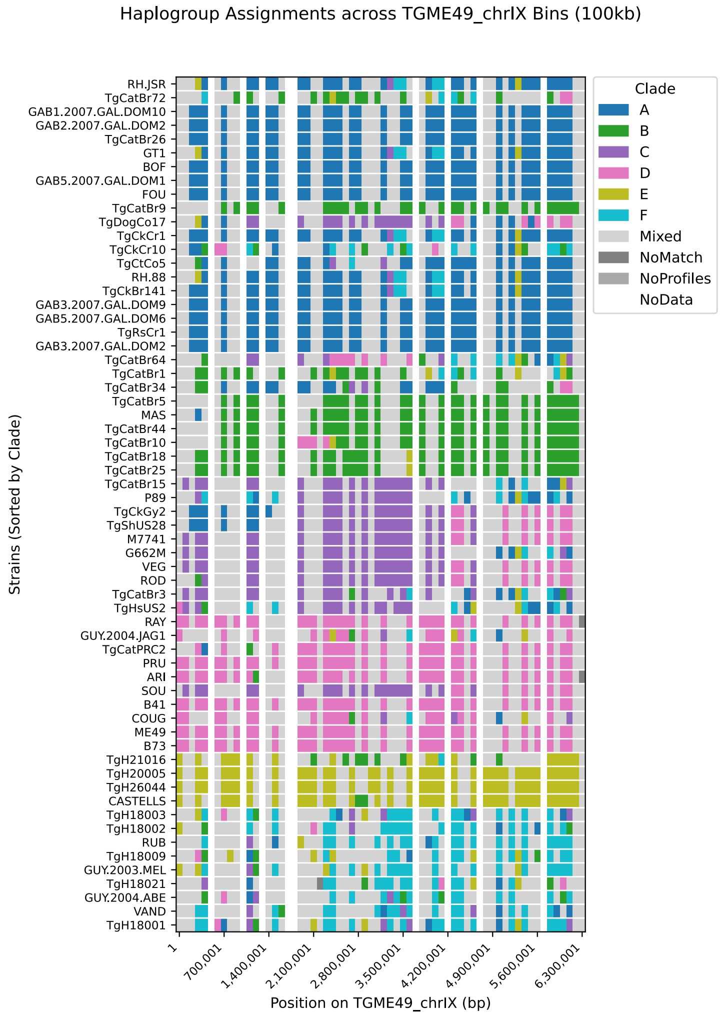
Haplogroup Assignments across TGME49_chrIX Bins (100kb)
RH.JSR
TgCatBr72
GAB1.2007.GAL.DOM10
GAB2.2007.GAL.DOM2
TgCatBr26
GT1
BOF
GAB5.2007.GAL.DOM1
FOU
TgCatBr9
TgDogCo17
TgCkCr1
TgCkCr10
TgCtCo5
RH.88
TgCkBr141
GAB3.2007.GAL.DOM9
GAB5.2007.GAL.DOM6
TgRsCr1
GAB3.2007.GAL.DOM2
TgCatBr64
TgCatBr1
TgCatBr34
TgCatBr5
MAS
TgCatBr44
TgCatBr10
TgCatBr18
TgCatBr25
TgCatBr15
P89
TgCkGy2
TgShUS28
M7741
G662M
VEG
ROD
TgCatBr3
TgHsUS2
RAY
GUY.2004.JAG1
TgCatPRC2
PRU
ARI
SOU
B41
COUG
ME49
B73
TgH21016
TgH20005
TgH26044
CASTELLS
TgH18003
TgH18002
RUB
TgH18009
GUY.2003.MEL
TgH18021
GUY.2004.ABE
VAND
TgH18001
Strains (Sorted by Clade)
1
700,001
1,400,001
2,100,001
2,800,001
3,500,001
4,200,001
4,900,001
5,600,001
6,300,001
Position on TGME49_chrIX (bp)
Clade
A
B
C
D
E
F
Mixed
NoMatch
NoProfiles
NoData

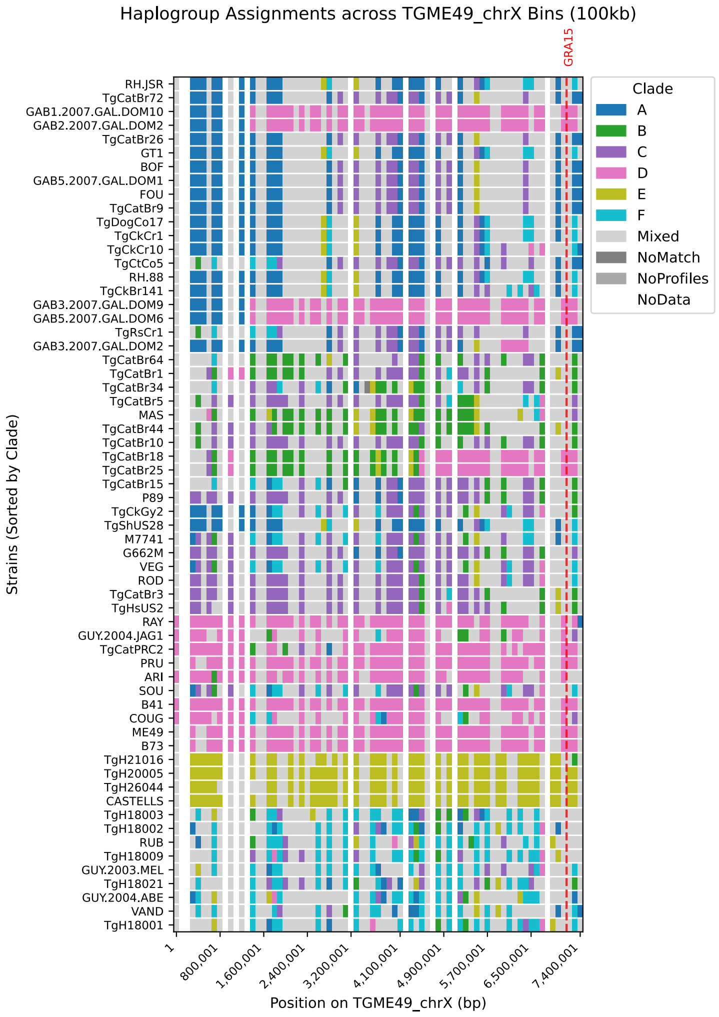
Haplogroup Assignments across TGME49_chrX Bins (100kb)
GRA15
RH.JSR
TgCatBr72
GAB1.2007.GAL.DOM10
GAB2.2007.GAL.DOM2
TgCatBr26
GT1
BOF
GAB5.2007.GAL.DOM1
FOU
TgCatBr9
TgDogCo17
TgCkCr1
TgCkCr10
TgCtCo5
RH.88
TgCkBr141
GAB3.2007.GAL.DOM9
GAB5.2007.GAL.DOM6
TgRsCr1
GAB3.2007.GAL.DOM2
TgCatBr64
TgCatBr1
TgCatBr34
TgCatBr5
MAS
TgCatBr44
TgCatBr10
TgCatBr18
TgCatBr25
TgCatBr15
P89
TgCkGy2
TgShUS28
M7741
G662M
VEG
ROD
TgCatBr3
TgHsUS2
RAY
GUY.2004.JAG1
TgCatPRC2
PRU
ARI
SOU
B41
COUG
ME49
B73
TgH21016
TgH20005
TgH26044
CASTELLS
TgH18003
TgH18002
RUB
TgH18009
GUY.2003.MEL
TgH18021
GUY.2004.ABE
VAND
TgH18001
Strains (Sorted by Clade)
1
800,001
1,600,001
2,400,001
3,200,001
4,100,001
4,900,001
5,700,001
6,500,001
7,400,001
Position on TGME49_chrX (bp)
Clade
A
B
C
D
E
F
Mixed
NoMatch
NoProfiles
NoData

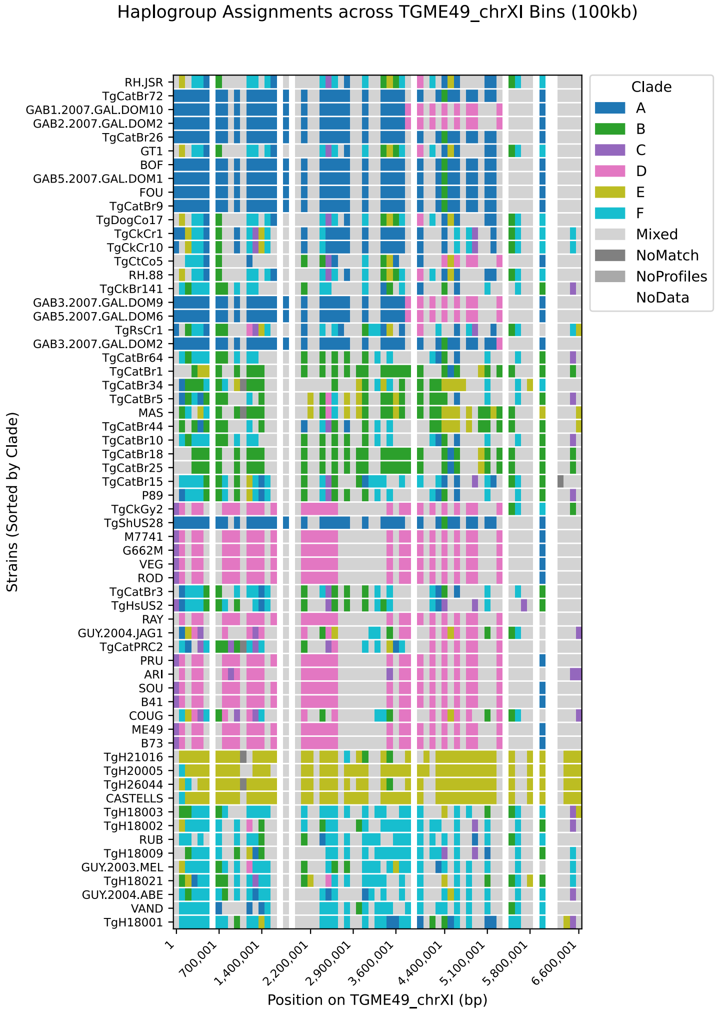
Haplogroup Assignments across TGME49_chrXI Bins (100kb)
Strains (Sorted by Clade)
RH.JSR
TgCatBr72
GAB1.2007.GAL.DOM10
GAB2.2007.GAL.DOM2
TgCatBr26
GT1
BOF
GAB5.2007.GAL.DOM1
FOU
TgCatBr9
TgDogCo17
TgCkCr1
TgCkCr10
TgCtCo5
RH.88
TgCkBr141
GAB3.2007.GAL.DOM9
GAB5.2007.GAL.DOM6
TgRsCr1
GAB3.2007.GAL.DOM2
TgCatBr64
TgCatBr1
TgCatBr34
TgCatBr5
MAS
TgCatBr44
TgCatBr10
TgCatBr18
TgCatBr25
TgCatBr15
P89
TgCkGy2
TgShUS28
M7741
G662M
VEG
ROD
TgCatBr3
TgHsUS2
RAY
GUY.2004.JAG1
TgCatPRC2
PRU
ARI
SOU
B41
COUG
ME49
B73
TgH21016
TgH20005
TgH26044
CASTELLS
TgH18003
TgH18002
RUB
TgH18009
GUY.2003.MEL
TgH18021
GUY.2004.ABE
VAND
TgH18001
1
700,001
1,400,001
2,200,001
2,900,001
3,600,001
4,400,001
5,100,001
5,800,001
6,600,001
Position on TGME49_chrXI (bp)
Clade
A
B
C
D
E
F
Mixed
NoMatch
NoProfiles
NoData

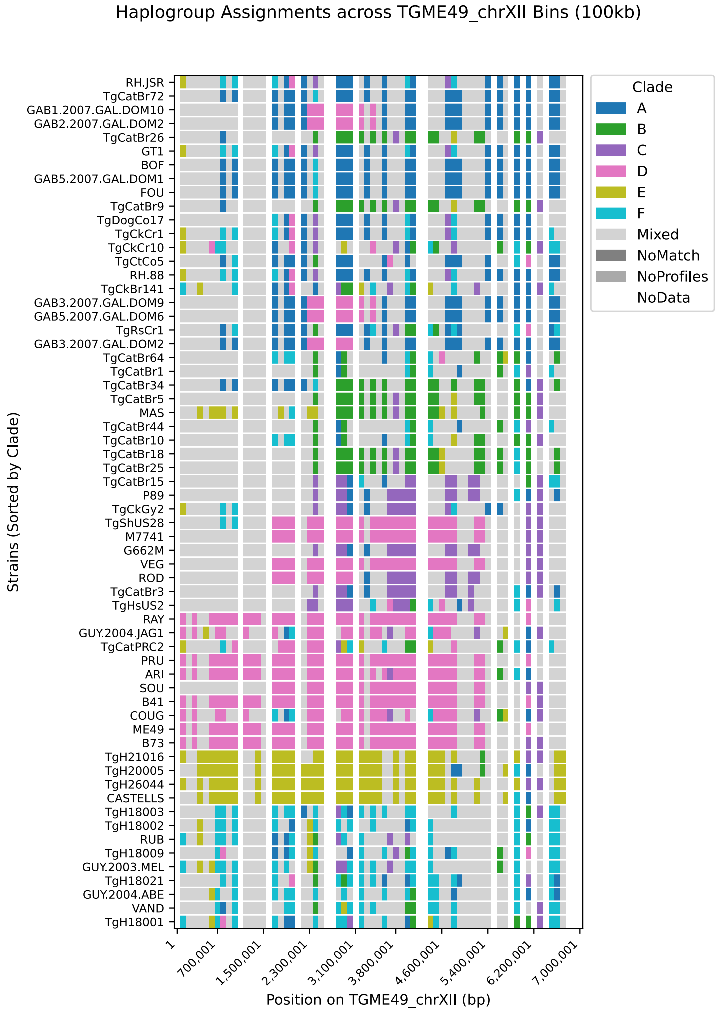
Haplogroup Assignments across TGME49_chrXII Bins (100kb)
Strains (Sorted by Clade)
RH.JSR
TgCatBr72
GAB1.2007.GAL.DOM10
GAB2.2007.GAL.DOM2
TgCatBr26
GT1
BOF
GAB5.2007.GAL.DOM1
FOU
TgCatBr9
TgDogCo17
TgCkCr1
TgCkCr10
TgCtCo5
RH.88
TgCkBr141
GAB3.2007.GAL.DOM9
GAB5.2007.GAL.DOM6
TgRsCr1
GAB3.2007.GAL.DOM2
TgCatBr64
TgCatBr1
TgCatBr34
TgCatBr5
MAS
TgCatBr44
TgCatBr10
TgCatBr18
TgCatBr25
TgCatBr15
P89
TgCkGy2
TgShUS28
M7741
G662M
VEG
ROD
TgCatBr3
TgHsUS2
RAY
GUY.2004.JAG1
TgCatPRC2
PRU
ARI
SOU
B41
COUG
ME49
B73
TgH21016
TgH20005
TgH26044
CASTELLS
TgH18003
TgH18002
RUB
TgH18009
GUY.2003.MEL
TgH18021
GUY.2004.ABE
VAND
TgH18001
1
700,001
1,500,001
2,300,001
3,100,001
3,800,001
4,600,001
5,400,001
6,200,001
7,000,001
Position on TGME49_chrXII (bp)
Clade
A
B
C
D
E
F
Mixed
NoMatch
NoProfiles
NoData
